# Estimating the abundance of the critically endangered Baltic Proper harbour porpoise (*Phocoena phocoena*) population using passive acoustic monitoring

**DOI:** 10.1002/ece3.8554

**Published:** 2022-02-19

**Authors:** Mats Amundin, Julia Carlström, Len Thomas, Ida Carlén, Jonas Teilmann, Jakob Tougaard, Olli Loisa, Line A. Kyhn, Signe Sveegaard, M. Louise Burt, Iwona Pawliczka, Radomil Koza, Bartlomiej Arciszewski, Anders Galatius, Jussi Laaksonlaita, Jamie MacAuley, Andrew J. Wright, Anja Gallus, Michael Dähne, Alejandro Acevedo‐Gutiérrez, Harald Benke, Jens Koblitz, Nick Tregenza, Daniel Wennerberg, Katharina Brundiers, Monika Kosecka, Cinthia Tiberi Ljungqvist, Ivar Jussi, Martin Jabbusch, Sami Lyytinen, Aleksej Šaškov, Penina Blankett

**Affiliations:** ^1^ Kolmarden Wildlife Park Kolmården Sweden; ^2^ AquaBiota Water Research Stockholm Sweden; ^3^ Centre for Research into Ecological and Environmental Modelling University of St Andrews St Andrews UK; ^4^ German Oceanographic Museum Stralsund Germany; ^5^ Marine Mammal Research Department of Bioscience Aarhus University Roskilde Denmark; ^6^ Chelonia Ltd Cornwall UK; ^7^ Turku University of Applied Sciences Turku Finland; ^8^ Prof. Krzysztof Skóra Hel Marine Station Department of Oceanography and Geography University of Gdańsk Hel Poland; ^9^ ProMare NPO Vintriku Saula küla, Kose vald Harjumaa Estonia; ^10^ Marine Research institute Klaipėda University Klaipėda Lithuania; ^11^ School of Biology Bute Building University of St Andrews St Andrews UK; ^12^ Ministry of Environment Helsinki Finland; ^13^ Department of Biology Western Washington University Bellingham Washington USA; ^14^ Present address: Department of Environmental Research and Monitoring Swedish Museum of Natural History Stockholm Sweden; ^15^ Present address: Department of Zoology Stockholm University Stockholm Sweden; ^16^ Present address: Max Planck Institute of Animal Behavior Konstanz Germany; ^17^ Present address: Centre for the Advanced Study of Collective Behaviour University of Konstanz Konstanz Germany; ^18^ Present address: Department of Biology University of Konstanz Konstanz Germany; ^19^ Present address: Swedish Meteorological and Hydrological Institute, Core Services Norrköping Sweden; ^20^ Present address: Scottish Association for Marine Science University of Highlands and Islands Oban UK; ^21^ Present address: County Administrative Board of Stockholm Stockholm Sweden; ^22^ Present address: Department of Biology ‐ Zoophysiology Aarhus University Aarhus Denmark; ^23^ Present address: Fisheries and Oceans Canada Maritimes, Dartmouth Nova Scotia Canada

**Keywords:** abundance estimation, C‐POD, detection function, passive acoustic monitoring, Phocoena phocoena

## Abstract

Knowing the abundance of a population is a crucial component to assess its conservation status and develop effective conservation plans. For most cetaceans, abundance estimation is difficult given their cryptic and mobile nature, especially when the population is small and has a transnational distribution. In the Baltic Sea, the number of harbour porpoises (*Phocoena phocoena*) has collapsed since the mid‐20th century and the Baltic Proper harbour porpoise is listed as Critically Endangered by the IUCN and HELCOM; however, its abundance remains unknown. Here, one of the largest ever passive acoustic monitoring studies was carried out by eight Baltic Sea nations to estimate the abundance of the Baltic Proper harbour porpoise for the first time. By logging porpoise echolocation signals at 298 stations during May 2011–April 2013, calibrating the loggers’ spatial detection performance at sea, and measuring the click rate of tagged individuals, we estimated an abundance of 71–1105 individuals (95% CI, point estimate 491) during May–October within the population's proposed management border. The small abundance estimate strongly supports that the Baltic Proper harbour porpoise is facing an extremely high risk of extinction, and highlights the need for immediate and efficient conservation actions through international cooperation. It also provides a starting point in monitoring the trend of the population abundance to evaluate the effectiveness of management measures and determine its interactions with the larger neighboring Belt Sea population. Further, we offer evidence that design‐based passive acoustic monitoring can generate reliable estimates of the abundance of rare and cryptic animal populations across large spatial scales.

## Graphical abstract

One of the largest ever passive acoustic monitoring studies has been carried out by eight Baltic Sea nations to estimate the abundance of the critically endangered Baltic Proper harbour porpoise for the first time. By logging porpoise echolocation signals at 298 stations during May 2011–April 2013, calibrating the loggers’ spatial detection performance at sea, and measuring the click rate of tagged individuals, an abundance of 71–1105 individuals (95% CI, point estimate 491) was estimated during May–October within the population's proposed management border. The small abundance strongly supports that the Baltic Proper harbour porpoise is facing an extremely high risk of extinction, and highlights the need for immediate and efficient conservation actions through international cooperation.

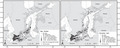

## 1 INTRODUCTION

Since its inception as a scientific discipline, a fundamental question in animal ecology is how many animals there are (Elton, 1927; Krebs, 1972). Based on repeated abundance estimates, trends can be inferred to determine the need for conservation actions and to estimate the efficacy of implemented conservation measures to ensure long‐term survival of a species, population, or management unit. However, abundance estimation is particularly challenging for marine mammals that migrate long distances, traverse national borders, and are visible only when they come to the surface to breathe. These challenges are further compounded when the population of interest is small and widely dispersed. As a result, many abundance studies of such species/populations rely on technological and statistical advances as well as integrated international efforts (Borowicz et al., 2019; Cubaynes et al., 2019; Guazzo et al., 2019; Hammond et al., 2013; Johnston, 2019).

**FIGURE 1:**
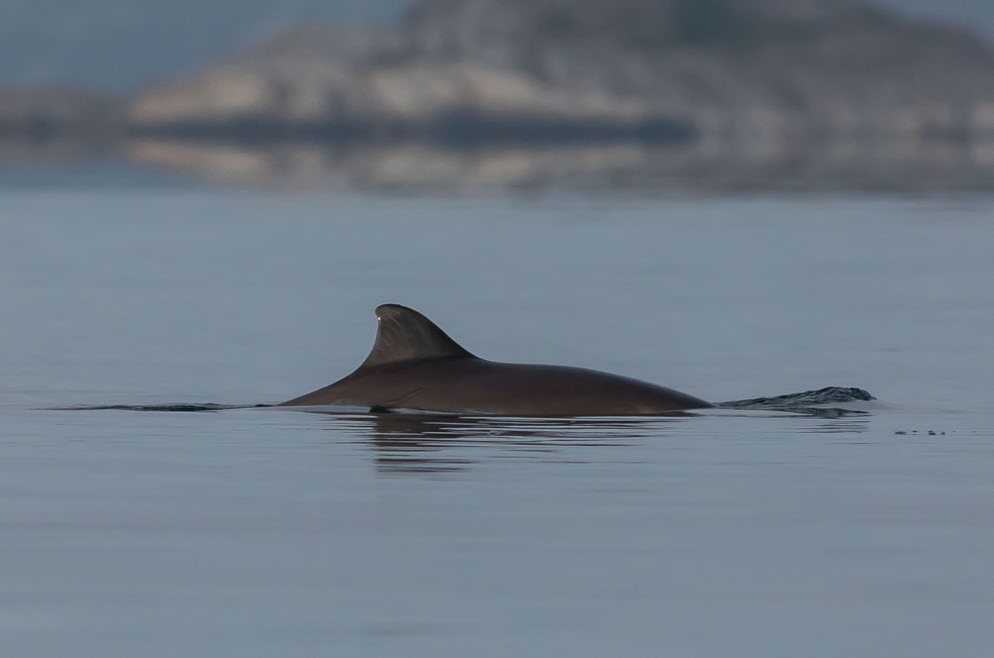
Harbour porpoise at the surface. Visual observations of the critically endangered Baltic Proper harbour porpoise are very rare. This animal was photographed at the Swedish west coast, where the species is more common. Photo: Håkan Aronsson

**FIGURE 2:**
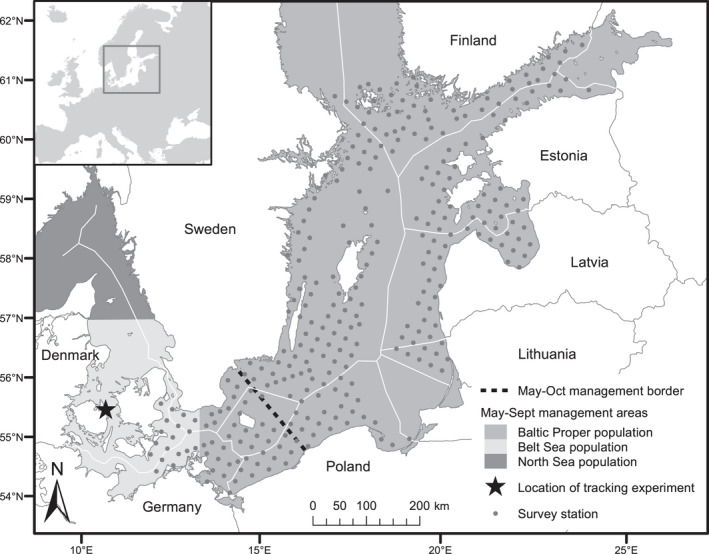
Proposed summer management borders of the harbour porpoise populations in the Baltic Sea and adjacent waters, and locations of the main survey stations and the tracking experiment in the SAMBAH study. The May–October management border has been proposed based on the spatial distribution of harbour porpoise in the southern Baltic Sea (Carlén et al., 2018). The shaded management areas have been proposed with focus on the abundance of the Belt Sea population (Sveegaard et al., 2015)

The harbour porpoise (*Phocoena phocoena*) (Figure 1) is the only resident cetacean species of the Baltic Sea, the world's largest body of brackish water. Two harbour porpoise populations use the Baltic Sea: (a) the Belt Sea population, inhabiting mainly the southern Kattegat, the Belt Sea including The Sound, and the southwestern Baltic Proper; and (b) the Baltic Proper population, inhabiting mainly the Baltic Proper (Carlén et al., 2018; Galatius et al., 2012; Lah et al., 2016; Sveegaard et al., 2015; Wiemann et al., 2010; Figure 2; Figure A4.1). Although the distributions of the Belt Sea and Baltic Proper populations are likely to overlap in winter, there seems to be a geographical separation between them during the reproductive season (Carlén et al., 2018). Based on this separation, a western management border of the Baltic Proper population during May–October has been suggested between the peninsula in Hanö Bay in Sweden and the village of Jarosławiec near Słupsk in Poland (Figure 2).

There is evidence of a drastic decline in numbers of harbour porpoises in the Baltic Proper since the mid‐20th century (Berggren & Arrhenius, 1995; Koschinski, 2001; Lindroth, 1962; Skóra & Kuklik, 2003). Bycatch in fishing gear has been identified as the most significant threat, and contaminant pollution as being of particular concern, in particular polychlorinated biphenyls (PCBs; Hammond et al., 2008; HELCOM, 2013). The distribution pattern of the Baltic Proper population has until recently been unknown (Carlén et al., 2018), and no population abundance estimate exists. However, the detection rate during dedicated surveys in the southern Baltic Sea has been very low (Berggren et al., 2004; Gillespie et al., 2005; Hiby & Lovell, 1996), and the Baltic Proper harbour porpoise has been listed as Critically Endangered (CR) by the IUCN since 2008 (Hammond et al., 2008) and by HELCOM since 2013 (HELCOM, 2013). The cryptic nature of the species, combined with its very low population density in the Baltic Proper, has precluded traditional survey methods such as mark–recapture via photographic identification or visual surveys by aerial or shipboard line transects. Aerial surveys were conducted in 1995 and 2002 (Berggren et al., 2004; Hiby & Lovell, 1996), observing a total of three and two single animals in an area covering the eastern part of the currently known management range of the Belt Sea population and the southwestern part of the currently known management range of the Baltic Proper population (Carlén et al., 2018; Sveegaard et al., 2015). The resulting abundance estimates are therefore not to be considered as population estimates.

During the last decade, passive acoustic monitoring methods have been developed to estimate the density and abundance of animals (Kyhn et al., 2012; Marques et al., 2013). The fundamental assumption is that detection rates of species‐specific sounds are a reliable proxy for animal density, once factors such as the detectability of the sounds are accounted for. Harbour porpoises vocalize nearly continuously for foraging, navigation, and communication (Akamatsu et al., 2007; Linnenschmidt et al., 2013; Wisniewska et al., 2016). Like all so‐called narrow‐band high‐frequency species, they generate sequences (“trains”) of powerful, directional, stereotypic, and narrow‐band high‐frequency clicks (Kyhn et al., 2013; Macaulay et al., 2020; Møhl & Andersen, 1973; Villadsgaard et al., 2007) in a frequency band where ambient noise is at a minimum (Richardson et al., 1995). These characteristics make the signals of narrow‐band high‐frequency species appropriate for passive acoustic monitoring, despite short detection ranges and a need for recorders with very high sample rate. In the Baltic Sea, the harbour porpoise is the only year‐round occurring cetacean species, and its signals can be safely distinguished from those of other sporadically occurring odontocetes.

Here, the eight EU Member States surrounding the Baltic Sea (Sweden, Finland, Estonia, Latvia, Lithuania, Poland, Germany, and Denmark) cooperated to conduct one of the largest passive acoustic monitoring studies to date in a joint effort, named Static Acoustic Monitoring of the Baltic Sea Harbour Porpoise (SAMBAH). The aim of the study was to estimate the density and abundance of the Baltic Proper harbour porpoise population for the first time.

## 2 MATERIALS AND METHODS

### 2.1 Survey area

The survey area encompassed the Baltic Sea from the Archipelago Sea around Åland in the north (south of 61°N) to the Darss sill (between Denmark and Germany, ca. 12°E) and the Limhamn/Drogden sill (between Sweden and Denmark, ca. 55° 50’N) in the southwest (Figure 2, Figure A4.2). The northern limit of the survey area was based on the current distribution of opportunistic sightings (HELCOM, 2022). The southwestern limit followed the definition that has been used in a previous study of the population structure of the harbour porpoise in the Baltic region (Berggren et al., 2002). The waters of the Exclusive Economic Zone of the Russian enclave, Kaliningrad Oblast, and the Russian waters in the eastern‐most part of Gulf of Finland were not included in the survey.

### 2.2 Main survey

#### 2.2.1 Survey design

The survey was designed to deploy approximately 300 acoustic data loggers throughout the study area (Figure 2). To achieve this, we created a randomly positioned and oriented systematic grid of survey locations (the “primary grid”) with a grid spacing of 23.5 km, distributed over the survey area in water depths between 5 and 80 m (for details, see Carlén et al., 2018). The depth data were obtained from the Baltic Sea Bathymetry Database (HELCOM, 2015). The 5‐m depth limit was set for safety reasons, that is, to make sure that boats would not hit the acoustic data loggers we deployed at each station (see below for details on the loggers), which were suspended with their hydrophones 2–3 m above the sea floor. Also, in shallower waters the loggers would be at higher risk during storms due to the wave action reaching down the bottom. The 80 m limit was chosen for two main reasons. This is the approximate depth of bottom areas with acute and permanent hypoxic conditions (<2 ml O_2_/l) in the Baltic Sea (Hansson & Andersson, 2015). Being an unsuitable bottom habitat for porpoise prey, low porpoise densities would be expected in these areas (Carlén et al., 2018). Further, an alternative rig design with acoustic data loggers suspended mid‐water to monitor pelagic porpoises would have required separate detection functions (see Auxiliary data collection below), deemed to be practically out of scope of this project. In a few cases, a logger could not be deployed at the primary location (e.g., due to military restrictions or shipping lanes). In these cases, if it was possible to find a tenable location within a few kilometers of the primary, this was used (average moved distance of nine stations was 3.3 km). If not, we chose at random location from the four closest secondary locations, where the grid of secondary locations (“secondary grid”) was offset 11.8 km from the primary grid (i.e., containing locations mid‐way between the primary locations) (Carlén et al., 2018). The final realized design (Figure 2) contains 304 sample locations (“stations”).

#### 2.2.2 Survey implementation

Our goal was to maintain a functioning acoustic data logger at each station for the full period of the survey, from May 1, 2011, to April 30, 2013. Logistical considerations meant that, in practice, some loggers were deployed before this period and some retrieved afterward. We excluded the data from outside the core period in all results presented here.

**FIGURE A1.1:**
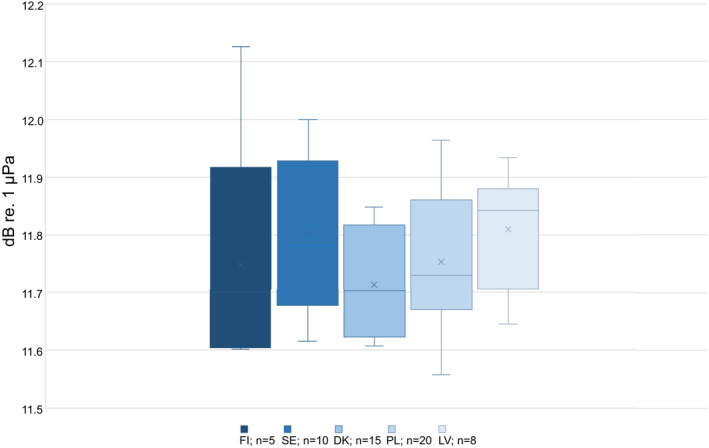
Detection thresholds for selected C‐PODs estimated by playback data. Detection threshold by country for a total of 58 selected C‐PODs; FI = Finland, SE = Sweden, DK = Denmark, PL = Poland, LV = Latvia. Whiskers, max and min; box, 25th and 75th quartiles; cross, mean; horizontal line, median

Acoustic data loggers were chosen instead of high‐frequency full‐bandwidth digital sound recorders, as such instruments were judged to be logistically infeasible. The logger used was the C‐POD (Chelonia Ltd.). The C‐POD is a click detector especially designed for logging very short, multi‐cycle signals such as the narrow‐band high‐frequency clicks generated by the harbour porpoise. C‐PODs are highly standardized to the same sensitivity by the manufacturer (Dähne et al., 2013). Some of the C‐PODs were also calibrated by SAMBAH personnel in a tank following the method described by Dähne, Gilles, et al. (2013) and Teilmann and Carstensen (2012), and some by using the received levels from the playback experiments (Appendix 1, Figure A1.1). Individual C‐PODs were rotated between stations to distribute any error caused by instrument variation.

#### 2.2.3 Acoustic processing

Since C‐PODs also log other sounds besides harbour porpoise clicks, the raw data were run through an adaptive classifier, the “KERNO” classifier, which is part of the C‐POD system (Tregenza, 2014). The classifier seeks “trains” of clicks in which successive clicks and inter‐click intervals resemble the previous and subsequent ones, and then gives each train a confidence class that the source is an actual train source, and assigns each train to a source type or “species.” For this study, an “encounter classifier,” called “Hel1,” was developed with the aim of minimizing the rate of false detections. Hel1 considers the trains of all “species” and the ambient noise, within encounters. An encounter runs from its first Hi or Mod quality NBHF train (defined by KERNO) to the last such train, with no gap between trains within the encounter being longer than 10 min. The resulting Hel1 classification makes no changes to the set of clicks forming the designated trains, but places all accepted trains into one quality class of possible harbour porpoise trains. In addition to processing the data by the classifiers, a subset of files with a low detection rate (equivalent of <60 detection positive minutes per year) was selected for visual inspection by trained experts, as this would most likely include all the files with no true positives. A total of 40,726 logging days were inspected, whereof the likely origin of false‐positive detections was noted for a subset of 22,689 logging days. Based on the duration of the visually inspected subset and the total dataset, and the assumptions that the spatial and temporal distribution of false positives was unrelated to porpoise detections, and that false positives were randomly distributed, we estimated a rate of 1 false detection positive minute per 247 recording days (see Appendix 2).

The acoustic results for each station were aggregated into 1‐second periods or “snapshots”; for each second, we recorded whether one or more harbour porpoise clicks were present or not. A minimum of five clicks are needed for KERNO, and following also Hel1, to classify a click sequence as a train. As we based our metric on Hel1 classified trains, the lowest number of clicks in a click‐positive second (CPS) was one. For trains beginning in one second and ending in a later second, all seconds from the beginning of the train until the end of the train were click‐positive (maximum inter‐click interval within a train of narrow‐band high‐frequency species is typically 250 ms; Tregenza, 2013). It was assumed that no more than one animal was recorded within each 1‐s snapshot. A longer time unit would have required estimates of group size, which are not available for the Baltic Proper (Berggren et al., 2002). To avoid interference from the servicing and the playback experiment, effort and click data from the days each C‐POD was deployed or retrieved were discarded.

### 2.3 Auxiliary data collection

Records of CPSs and survey effort seconds, both obtained from the main survey, are not sufficient on their own to estimate absolute density or abundance: we also need to know the area surveyed by the loggers (Marques et al., 2013). The probability of logging one or more clicks from a harbour porpoise over a 1‐s period is, on average, a decreasing function of its horizontal distance from the sensor. Many other factors are also important, such as whether the harbour porpoise is clicking or not, the direction and depth of its swimming, and the sonar beam scanning behavior. We therefore used a concept from the distance sampling survey literature (e.g., Buckland et al., 2001): the effective detection area (EDA). In the current context, the EDA is the area of a horizontal circle centered on the logger within which, on average, as many harbour porpoises are missed in a 1‐second period as are detected outside the circle. (Note that we work in 2 dimensions, rather than 3, by projecting onto the horizontal plane—i.e., animal density is per unit area of water, not volume; variation in EDA caused by differences in water depth are captured to some extent by including depth as a covariate in the playback experiment analysis, see below.)

We used three auxiliary studies to estimate the EDA by month and location. First, the “tracking experiment”: in an area of relatively high porpoise density (necessarily outside the survey area), we acoustically tracked porpoises in the vicinity of C‐PODs to determine the per‐second probability of detection as a function of horizontal animal‐logger distance. This experiment yielded estimates of EDA for clicking porpoises in one location during summer. Second, the “tagging study”: we used data from six porpoises fitted with acoustic recording tags to estimate the proportion of time porpoises are in a non‐clicking (i.e., silent) state. Third, the “playback experiment”: we undertook playbacks of artificial porpoise click trains over a range of distances away from the C‐PODs at both the tracking experiment site and most sampling locations in the main study. This allowed us to determine how distance‐specific detection probability changed as a function of environmental factors, and hence generalize our results from the location and time of the tracking experiment to estimate EDA for all locations and months surveyed. Below each of these studies are described in detail. We then describe the statistical analyses that combined the results from these auxiliary studies with those from the main survey to yield estimates of porpoise density and abundance.

#### 2.3.1 Tracking experiment

A challenge in using passive acoustics to detect harbour porpoises is that their echolocation signals are highly directional (Au et al., 1999; Koblitz et al., 2012; Macaulay et al., 2020), and they may adapt their source levels to different acoustic habitats (Dähne et al., 2020). Although the directionality is partly compensated by the scanning movements of the head performed by harbour porpoises (Verfuss et al., 2009), the combined effect of click directionality, source level, head‐scanning behavior, and general swim direction on the detectability of harbor porpoises needs to be measured empirically. We estimated the EDA of a C‐POD by acoustically tracking free‐ranging harbour porpoises with hydrophone arrays in an area where C‐PODs were moored to the seabed.

This experiment was undertaken from May 27 to June 22, 2013, in the Great Belt, Denmark (Figure 2), at a water depth of 19.5 m. This site (55° 27.2’ N, 10° 50.6’ E) was selected because porpoise density was known to be high enough to yield a useable number of porpoise encounters in the time available for the experiment; the low density of porpoises in the main part of the survey area prevented us from conducting the experiments there. A harbour porpoise‐tracking hydrophone array was constructed and attached to a 12.5‐m research vessel. A horizontal array consisted of a cross of five hydrophones, two in port‐starboard and three in bow‐stern orientation. The recordings made with the horizontal array allowed us to obtain the bearing of the animal relative to the array. In addition, we deployed a vertical array with an aperture of 13 m consisting of 10 evenly spaced hydrophones tied to a rope with a 100 kg weight at the bottom end (well above the sea floor) to assure the straight vertical orientation. The vertical array was used to determine distance and depth of the echolocating harbour porpoises. Combining this with the accurate GPS position of the boat and measuring the boat's orientation allowed us to reconstruct the geo‐referenced positions from which all clicks were emitted and resulted in a swim path of the animal.

At the study site, 16 C‐PODs were moored with the hydrophone approximately 2 m off the seabed in a 4x4 grid with 50 m spacing. The vessel with the arrays was anchored both by the bow and the stern at a corner of the grid. OpenTag™ inertial measurement units (Loggerhead Instruments) were placed on the array at regular intervals, measuring its 3D underwater orientation (for further details, see Macaulay et al., 2017). A vector GPS and an OpenTag™ unit were placed on the boat to precisely measure the track and heading of the vessel and its tilt and roll. In addition to the acoustic tracking of harbour porpoises swimming in the area, two visual observers were placed on the wheelhouse of the survey vessel during daylight hours. The observers scanned a sector of 180° each, recording the time, bearing, distance, and number of animals of each sighting. Since click trains from different porpoises cannot be distinguished in C‐POD data, only encounters where we were confident that a single animal was present, based on the acoustic tracking data alone or in combination with the visual data, were used in the analysis—these encounters are referred to as “tracking events.”

Through the hydrophone array, the full frequency bandwidth of the animals’ click trains was recorded on a computer, using a custom‐made software called Malta (Microphone Array Localisation Tool for Animals). Acoustic data from the tracking array and the spatial data of the OpenTag™, the roll and tilt sensors, and the GPS were post‐processed using the PAMGUARD (https://www.pamguard.org/) and MATLAB (MathWorks Ltd). The time‐of‐arrival differences from a click detected on multiple hydrophones were used to calculate the instantaneous geo‐referenced 3D position of a harbor porpoise. As the porpoise swam through the survey area, multiple click positions were used to reconstruct the 3D animal tracks. These tracks were used to give an estimate of the animal's position each second and hence the horizontal distance from the harbour porpoise to each C‐POD. C‐POD data were processed in the same way as data from the main acoustic survey to yield CPS, and these were time‐matched to the swim tracks. A strong diurnal pattern in detectability was noted, and each tracking event was classified into whether it occurred during dawn, day, dusk, or night. Dawn is the time between beginning of civil twilight and sunrise, and dusk the time between sunset and end of civil twilight. The start and end times of the diel phases were obtained from the United States Naval Observatory (2013). The diel phase was then used as a factor in the data analysis. For the five days with porpoise tracks, the average length of dawn and dusk was nearly 2 hours, respectively, of day 15 h 24 min, and of night 4 hours 40 min.

#### 2.3.2 Tagging study

The tracking experiment described above is capable of yielding a detection function (and hence EDA) for clicking harbor porpoises. However, it was unknown if harbour porpoises click all the time, something that must be taken into account. To this end, six individuals that were incidentally entrapped in Danish fixed pound nets were fitted with acoustic and depth recording tags (Wright et al., 2017). As the animals were in‐hand when the tag was attached, each tag could be located in a near‐identical position on the dorsal fin for greatest consistency across the datasets. The acoustic tag was a second‐generation A‐tag (ML200‐AS2: Marine Micro Technology, Saitama, Japan; see (Kimura et al., 2013)), which is a click event logger with two hydrophones placed 105 mm apart, in line with the body axis of the animal. The tag stores the sound pressure level and the time stamp of each received click. The hydrophone detection threshold is 133 dB (peak‐to‐peak) re 1 µPa within a frequency range of 55–235 kHz. Neither waveform nor duration of the clicks was recorded. The time‐of‐arrival difference between the two hydrophones makes it possible to calculate the bearing to the source and was used to separate sounds generated by the tagged animal from those of other porpoises in the vicinity (see Wright et al. (2017) and references therein). The depth recorder (DST‐Milli‐F logger, Star‐Oddi, Iceland) had a 1‐m resolution and was set to log data at 3‐s intervals. The tags remained attached for multiple days and were recovered by Argos and VHF tracking once detached from the animal using a timed releaser (Wright et al., 2017).

The acoustic records were processed to yield click times, and these were aggregated into CPSs. The tags were programmed to duty cycle, typically recording for 10 min each hour. Data from the first two hours after release were discarded, as were data from seconds where the animal was <2 m from the water surface (as estimated for each second by linear interpolation between the 3‐second samples of the depth records). The acoustic depth truncation was necessary because there was too much acoustic interference from the surface, such as wave noise, surface reflections, and breathing, for the tag to reliably detect the echolocation clicks generated by the tagged animal. The resulting data were analyzed to produce estimates of the average probability of the tagged animal producing one or more CPS during periods of time equal to a tracking event in the harbor porpoise‐tracking experiment (see Tracking experiment above and Statistical analyses below).

#### 2.3.3 Playback experiment

The datasets from the tracking and tagging experiments can be used to estimate the EDA of harbor porpoises in the Great Belt at the time of the tracking experiment. However, this may not apply to the main acoustic survey if harbour porpoise behavior influencing their acoustic detectability (hereafter referred to as “acoustic behaviour”), or the acoustic propagation, changes over space, depth, or time. We could not account for variation in acoustic behavior, but to account for propagation differences we conducted playbacks of artificial harbour porpoise click sequences both in the Great Belt during the tracking experiment and at a sample of survey stations during the main survey.

Playbacks were conducted using omni‐directional piezo‐electric transducers (Denmark and Germany: TC4033, Reson A/S, Slangerup, Denmark; Sweden, Finland, Estonia, Latvia, Lithuania, and Poland: HS/150, Sonar Research & Development, Beverly, UK), suspended to a depth of ca. 5 m, at a range of up to 8 horizontal distances from the deployed C‐POD, designed to span 0–500 m. Each playback consisted of a set of 11 artificial harbour porpoise‐like click sequences, and each sequence consisted of 10 or 20 equally spaced clicks with an inter‐click interval of 1 ms. The inter‐sequence interval was 10 or 50 ms. The artificial clicks were a 100 ms pure tone at 130 kHz, shaped by a raised cosine (Hann window). The playback signals were generated by a laptop computer connected to a National Instruments D/A‐converter (DAQPad 6070E, USB‐6251 or USB‐6361) and amplified by an A‐301 HS High Voltage piezo amplifier (AA Lab Systems, Tel Aviv). The designed peak‐to‐peak source level (SL_p‐p_) for the first click sequence was 186 dB re 1 µPa m, with each subsequent click sequence reduced by 3 dB, resulting in the final sequence having a SL_p‐p_ of 156 dB re 1 µPa m (unit defined as in Ainslie, 2011). However, on reviewing the recordings of the playbacks made in proximity to the source, it was discovered that playbacks with the TC4033 transducer were limited in peak–peak level due to system overload for source levels greater than 181 dB re 1 µPa m. For the HS/150 transducer, the limitation was for levels above 169–171 dB re 1 µPa m (measured at two different occasions). This resulted in the highest usable SL_p‐p_ of 168 dB re 1 µPa m for all playbacks; click sequences with a SL_p‐p_ at or above 171 dB re 1 µPa m were excluded from further analysis. Playbacks were performed with the vessel's engine and echo sounder switched off.

After recovery of the C‐PODs, time periods corresponding to the playback were examined and, for each artificial click sequence, the number of clicks that were detected (out of either 10 or 20 clicks) for a given source level and distance was recorded. Note that most of the time periods for the playbacks were discarded from the main dataset to not interfere with surveyed effort or click data.

### 2.4 Statistical analyses

Here, we describe the estimation of harbour porpoise density and abundance, then the analyses associated with each part of the density formula, and, finally, variance estimation. All analyses were performed using the statistical software R version 4.1.1 (R Core Team, 2021). Further details are provided in the R Sweave files placed in the Dryad repository associated with this article (see Data accessibility statement).

#### 2.4.1 Porpoise density and abundance

Porpoise density was initially estimated separately for each sampling location, month, and diel phase (dawn, day, dusk, and night, calculated using sunrise and sunset times for the 15th day of the month at each location), as follows:

(1) $${\hat{D}}_{\text{imd}}=\frac{n_{\text{imd}}}{T_{\text{imd}}{\hat{\nu }}_{\text{imd}}}$$

where *D* is density, *n* is the number of CPS, *T* is the number of seconds of monitoring effort, ν is the EDA, the hat symbol ^ indicates an estimate, and subscripts *imd* indicate that all quantities are for sampling location *i* in month *m* and diel phase *d* (1 = dawn, 2 = day, 3 = dusk, 4 = night). We return to the estimation of ν below (see Effective detection area (EDA), below). Density per sampling location and month was estimated as a weighted mean of the diel phase density estimates:

(2) $${\hat{D}}_{\text{im}}=\sum_{d=1}^{4}w_{\text{imd}}{\hat{D}}_{\text{imd}}$$

where $w_{\text{imd}}$ is the proportion of the 15th day of month *m* at location *i* that is made up of diel period *d*. Density was aggregated to the level of season and country within region (northeast or southwest of the proposed management border shown in Figure 2 as the mean of the relevant location‐ and month‐specific estimates). For this purpose, Denmark Bornholm was treated as a separate “country” from other Danish waters. Density by region was calculated as a survey area weighted mean of the relevant country‐by‐region estimates. Abundance was estimated as density multiplied by survey area.

#### 2.4.2 Effective detection area (EDA)

The EDA for each sampling location, month, and diel phase was estimated as:

(3) $${\hat{\nu }}_{\text{imd}}=\frac{{\hat{\nu }}_{d}^{*}{\hat{p}}_{c}{\hat{\xi }}_{\text{im}}}{{\hat{\xi }}^{*}}$$

where ${\hat{\nu }}_{d}^{*}$ is the estimated EDA for harbour porpoises in diel phase *d* estimated from the tracking experiment; ${\hat{p}}_{c}$ is the estimated probability that harbour porpoises produce one or more clicks during the time period of a tracking event in the tracking experiment—this is estimated from the tag data; ${\hat{\xi }}^{*}$ is the predicted EDA for an artificial click at the tracking experiment site in the Great Belt, estimated from the playback experiment at that location; and ${\hat{\xi }}_{\text{im}}$ is the predicted EDA for an artificial click at sampling location *i* and month *m*, estimated from the playback experiment in the main survey area.

The motivation for this formulation is as follows. The tracking experiment enables estimation of $\nu_{d}^{*}$, the EDA for harbour porpoises that were clicking and therefore available to be tracked acoustically and take part in the experiment. However, the EDA required is for clicking and non‐clicking harbour porpoises, which is estimated by ${\hat{\nu }}_{d}^{*}{\hat{p}}_{c}$. To generalize this EDA to apply to sites within the main survey area, we assume that the ratio of EDA for artificial clicks from playbacks at the tracking experiment site ($\xi^{*}$) to EDA of artificial clicks at a main survey site ($\xi_{\text{im}}$) is equal to the ratio of true harbour porpoise EDA at the tracking location site in any diel phase ($\nu_{d}^{*}p_{c}$) to the true harbour porpoise EDA at the main survey site in the same diel phase ($\nu_{\text{imd}}$) – that is,

(4) $$\frac{\xi^{*}}{\xi_{\text{im}}}=\frac{\nu_{d}^{*}p_{c}}{\nu_{\text{imd}}}$$

yielding Equation 3.

We now describe the analyses used to estimate $\nu_{d}^{*}$ from the tracking experiment, $p_{c}$ from the tagging study, and $\xi^{*}$ and $\xi_{\text{im}}$ from the playback experiment.

#### 2.4.3 Analysis of the tracking experiment

The goal was to estimate the EDA, $\nu_{d}^{*}$, given input data consisting of, for each tracking event, the estimated horizontal distance of the harbor porpoise from each C‐POD in each second of the event, and whether the C‐POD detected clicks or not (after processing with the KERNO and Hel1 classifiers). Each second on each C‐POD during a tracking event forms a binary trial, with a “success” being detection of clicks and a “failure” being non‐detection. We therefore analyzed the data using binary regression, with detection/non‐detection as the response variable, distance and diel phase as continuous and factor covariates, respectively, and a logit link function. Our approach was similar to that of Kyhn et al. (2012), except that we did not assume a linear‐logistic shape for the detection function (the relationship between detection probability and distance). Instead, we used a Generalized Additive Model (GAM, Wood, 2017) to allow a smooth, nonlinear relationship between probability of detection and distance. We used cubic regression spline bases; initial fits produced implausible shapes due to the patchy distribution of distances in some diel phases and the very small proportion of successes, so we hand‐selected only three knot points (at 100, 300, and 500 m) to ensure a smooth, nonlinear function. Given the very conservative click classifier used, detection probability can be safely assumed to be zero at 500 m; this constraint was added to the model adding structural zeros to the data at 500 m so that estimated detection probability was zero at that distance with no uncertainty. Fitting was implemented using the package mgcv in R (Wood, 2017).

Trials within the same second are not independent between C‐PODs, and trials within the same tracking event are not independent—this will have a negligible effect on the estimated functional relationship but can strongly affect variance. To account for this effect, we used a non‐parametric bootstrap (using tracking event as the sampling unit) to estimate variance (see Variance estimation below).

Given the fitted detection function from the GAM, we used the following formula to give an initial estimate of EDA for each diel phase—it is based on the point transect formulae of Buckland et al. (2001); see also Kyhn et al. (2012) (although that paper uses effective detection radius rather than EDA):

(5) $${\hat{\nu }}_{d}^{**}=2\pi \int_{r=0}^{w}r\hat{g}\left(r,d\right)dr$$

where $\hat{g}\left(r,d\right)$ is the estimated detection function for horizontal distance *r* and diel phase *d*, and *w* is some horizontal distance at which detection probability is assumed to be zero. We used *w *= 500 m.

In practice, the sample size of tracking events in each diel phase was small (4 in the morning phase, 21 in the day, 5 in the evening, and 6 in the night), severely limiting our ability to infer accurately diurnal changes in porpoise detectability from the above analysis. Also, it is possible that diurnal behavior was different here from other parts of the Baltic (see Discussion). We therefore used information from the main acoustic survey to inform our estimate of the relative detectability of porpoises by diel phase, as follows. The basic idea is that the number of porpoises present within each country and month does not vary by diel phase, and hence changes in porpoise detection rate by diel phase within country and month must be due to changes in detectability. We therefore fitted a statistical model of detection rate as a function of diel phase (with day as the base level) plus the interaction of month (as a factor) and country. We used a Generalized Linear Model (GLM) with detection rate modeled as a Tweedie random variable (Tweedie, 1984) to accommodate for overdispersion relative to a Poisson variable, and using a log link function. The estimated diel phase coefficients were exponentiated to yield estimates of proportional change in detection rate (and hence, by assumption, in detectability) by diel phase, relative to the day phase—we denote these $e_{d}$. The EDRs calculated from Equation 5 were then scaled as follows:

(6) $$\nu_{d}^{*}=\frac{e_{d}\sum_{d=1}^{4}w_{d}^{*}\nu_{d}^{**}}{\sum_{d=1}^{4}w_{d}^{*}e_{d}}$$

where $w_{d}^{*}$ is the proportion of the day at the tracking experiment site that is made up of diel period *d* (equal to 0.084, 0.660, 0.084 and 0.171 for dawn, day, dusk and night respectively). The scaled EDRs, $\nu_{d}^{*}$, thus have the same weighted average (weighted by $w_{d}^{*}$) as the unscaled ones ($\nu_{d}^{**}$), but their relative magnitude is the same as the $e_{d}$s, so relative detectability matches that found from the main survey area. These scaled EDRs were used in Equation 3.

#### 2.4.4 Analysis of tagging study

Our goal was to estimate $p_{c}$, the average probability of one or more CPS during a period of time equivalent to the length of the tracking events in the tracking experiment. Input data were, for each tagged harbour porpoise, the presence or absence of a click for each second of recording where the harbour porpoise was estimated to be deeper than 2 m (acoustic data from depths <2 m had been removed, see Tagging study above). Data from each tagged harbour porpoise were analyzed separately. Within this, we undertook a separate analysis for each tracking event duration from the tracking experiment. For each of the 36 harbour porpoise‐tracking events, we divided the tag record into chunks of that duration. Only chunks where the tag was recording for the entire duration of the chunk were retained (recall that the acoustic recorder was duty cycled). The mean tracking event duration was 64 s (maximum 263 s) so given a typical duty cycle of 10 minutes this meant only discarding a small proportion of chunks. For the remaining chunks, we recorded whether the chunk contained any CPS and the proportion of the chunk where depth was <2 m – that is, of missing click data. To correct for the missing data, we fitted a binary regression of the presence/absence of at least one CPS vs. a monotonic non‐increasing smooth function on the logit scale of the proportion of missing data (using the package scam in R (Pya & Wood, 2015)), and predicted the probability of one or more click for zero missing data. Let ${\hat{p}}_{\text{cae}}$ be the predicted probability of there being at least one CPS for tagged animal *a* and tracking event duration *e*. We estimated average probability of one or more CPS for each tagged animal, ${\hat{p}}_{\text{ca}}$, by taking the mean across all tracking event durations. Finally, we estimated the overall average probability of one or more CPS, ${\hat{p}}_{c}$, by taking a weighted mean of ${\hat{p}}_{\text{ca}}$ over all tagged animals, weighting by the number of seconds that each animal's tag was recording and the animal was deeper than 2 m.

#### 2.4.5 Analysis of playback experiment

The goal was to estimate the EDAs $\xi^{*}$ and $\xi_{\text{im}}$ for the Great Belt tracking experiment and all stations and months in the main survey area. The two datasets (tracking experiment location and main survey area playbacks) were analyzed separately. Input data variables for both were detection/non‐detection of each click within an artificial click sequence, together with horizontal distance and playback source level. In addition, for the main survey playbacks, a set of candidate environmental, spatial, and temporal variables that potentially affect sound propagation were obtained for each month and station. These included sediment type, depth (m), temperature (^o^C), salinity (PSU), pycnocline depth (m), pycnocline gradient (kg/m^3^/m), date (year and month or Julian day), and location (latitude and longitude) (see Table A5.1 for full details). Oceanographic variables were acquired from the Swedish Meteorological and Hydrological Institute (SMHI). They were derived from an oceanographic model at the spatial resolution of 0.083 decimal degrees and temporal resolution of one month. Depth was derived from the Baltic Sea Bathymetry Database at the resolution of 500 × 500 m (HELCOM, 2015). Sea‐surface salinity had a few unusually high values so to increase model robustness we trimmed the highest 1%, setting them equal to the 99th percentile value.

Separate models were fitted to each dataset. Both were binary GAMs, implemented using the package mgcv in R (Wood, 2017), with detection/non‐detection of each click as response variable, and covariates modeled via a logit link. Both models included distance and source level as smooth continuous covariates; model selection showed that modeling these jointly as an interaction (a tensor product of cubic regression splines) produced a better fit (lower AIC). For the main study playback analysis, additional covariates were selected for inclusion in the model that were not highly correlated with one another (|*r*| < .5) and were modeled as main effects without consideration of interaction terms. Sediment type was modeled as a factor covariate, month, or Julian day as cyclic regression splines and the other variables as thin‐plate regression splines. In all cases (except the tensor product), to avoid unrealistically complicated models, smooth functions were limited to a maximum of 5 degrees of freedom. Variables were added by forward selection, with those resulting in a lower AIC being retained. Environmental variables (e.g., depth and sediment type) were offered for inclusion before explicitly temporal (e.g., month) or spatial (e.g., latitude and longitude) variables (see Table A5.1).

The selected models were used to estimate EDA, by integrating out distance in a similar way to Equation 5. A single source level was used—we selected to use SL_p‐p_ of 168 dB re 1 µPa m, the highest level consistently used in the Great Belt playbacks, it being the closest we could come to the nominal on‐axis source level of a harbor porpoise (cf. Villadsgaard et al. (2007)), who report SL_p‐p_ of 178–205 dB re 1 µPa m). For the main study, values of the environmental covariates were sometimes outside the range of those used to fit the model; in these cases, to avoid extrapolation, we constrained them to lie within the range of values for the stations where playbacks took place.

There are several levels of potential non‐independence in the playbacks. Clicks at a given source level are not independent within a playback; in the main survey, playback hardware is not independent between stations and C‐PODs were re‐used at multiple stations; in the Great Belt study, each playback was broadcast to multiple C‐PODs. For the main survey study, we implemented variance estimation via a non‐parametric bootstrap, with the sampling unit being a playback session (i.e., a set of playbacks at a station on the same date). We note that model selection is also affected by non‐independence, and hence, it is possible that we selected a model with too many explanatory variables; this will not lead to bias but will reduce precision. For the Great Belt tracking experiment, there were few playback sessions, so we instead included in the model a random effect for playback and another for C‐POD (implemented via the re smoother in the mgcv package (Wood, 2017)). Variance estimation in this case was implemented via a parametric bootstrap, using the fitted model coefficients and associated variance–covariance matrix and assuming the coefficients follow a multivariate normal distribution.

#### 2.4.6 Variance estimation

Variance and confidence interval estimation were implemented via a bootstrap procedure, where each component of the density (and abundance) estimate was generated from an independent bootstrap, as follows. For detection rate (*n* and *T*), a non‐parametric bootstrap was used, resampling sampling locations within country within region. (One issue was that there was only one sampling location in the northeast region of Danish Bornholm so no variance could be computed in this stratum. However, since the abundance in this stratum was zero in May–October and two in November–April, the lack of variance had a negligible effect in practice.) For the acoustic tracking experiment EDA, $\nu_{d}^{*}$, a non‐parametric bootstrap was used, resampling harbour porpoise‐tracking events within diel phase (in re‐fitting the models, structural zeros were used to ensure that all fitted functions had an estimated detection probability of 0 at 500 m). For the tagging study, a parametric bootstrap was used, because there were too few tagged animals for a non‐parametric bootstrap. The estimated average probability of one or more CPS, ${\hat{p}}_{c}$, and its associated variance, were fitted to a beta distribution by matching the first two moments. Random samples were then generated from this distribution to produce bootstrap realizations of $p_{c}$. For the playback EDA at Great Belt, $\xi^{*}$, a parametric bootstrap was used, resampling from the fitted detection function model. For the playback EDAs in the main study, $\xi_{\mathrm{im}}$, a non‐parametric bootstrap was used instead, resampling playback sessions, but ignoring model selection uncertainty (i.e., using only the final model selected in analysis of the original dataset rather than re‐implementing model selection within the bootstrap).

In all cases, 1000 bootstrap resamples were generated. For each bootstrap replicate, harbour porpoise density at each site and month was estimated, using Equations (1), (2), (3), (4), (5), (6); these site and month estimates were then combined as described in the section Density and abundance above, to produce 1000 bootstrap replicate estimates of density and abundance at the level of seasons and region. Estimates of variance in density and abundance were derived from the bootstrap replicates using the standard estimator of variance, and confidence intervals were derived using the percentile method (see Kyhn et al., 2012).

#### 2.4.7 Assumptions

We here summarize the assumptions used in estimating abundance. (1) At most one individual porpoise is detected in each one‐second snapshot at each location. (2) There are no false‐positive detections. (3) Porpoise density at sampling locations within each country and region is representative of the density in that country and region. (4) Missing C‐POD data at sampling locations are missing at random within location and month. (5) Only single porpoises were part of the Great Belt tracking experiment. (6) Acoustic behavior of porpoises in the Great Belt tracking experiment is representative of acoustic behavior of porpoises in the main survey area. (7) Animals with acoustic tags have temporal click patterns representative of animals within both the Great Belt and the main study area. (8) The temporal pattern of clicks in sections of the tag record that are missing is the same, on average, as that in the sections we used for analysis. (9) The statistical models used to estimate EDA of porpoises in the trials at the Great Belt, and EDA of playbacks at Great Belt and in the main survey area, produce unbiased estimates.

In deriving estimates of uncertainty (variance and confidence intervals), we made the following additional assumptions. (10) The sampling locations are located independently and at random within region within country. (11) Porpoise‐tracking events in the Great Belt tracking experiment are independent of one another. (12) The beta distribution fitted to the estimate of proportion of time clicking from the tagging study accurately represents uncertainty on that parameter. (13) The model used to estimate EDA of playbacks in the Great Belt study produces an unbiased estimate of parameter variance and covariance; parameters follow a multivariate normal distribution. (14) Playback sessions in the main survey area are independent.

## 3 RESULTS

### 3.1 Survey effort

**FIGURE 3:**
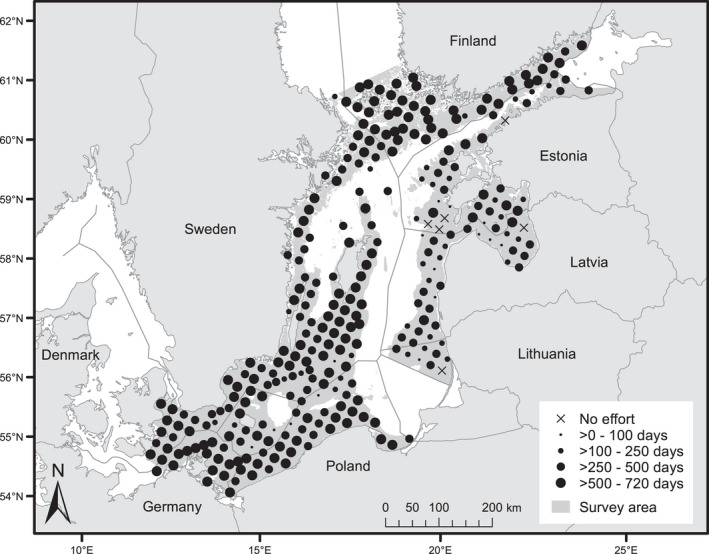
Recording effort per station May 2011–April 2013. The radius of each dot is proportional to the number of days of survey effort; crosses are stations with no survey effort. The shading shows the main survey area

During the survey period from May 1 2011 to April 30, 2013, C‐POD click loggers were deployed and data were successfully retrieved from 298 of the designed 304 survey stations (Figure 3). The recorded data corresponded to a total of 377 logging years, representing 62% of the total possible effort if all 304 stations had been active for the entire two‐year survey period. There was strong spatial variation in effort, with considerably lower effort primarily in Estonia, Latvia, and Lithuania (Figure 3). There, loggers were removed by trawling and the coast is very exposed to foul weather and ice, which interfered with servicing to exchange batteries and memory cards. There was also temporal variation in effort, with lower survey coverage in late 2011 and early 2012 (Figure A4.2).

### 3.2 Acoustic detection rates

**FIGURE 4:**
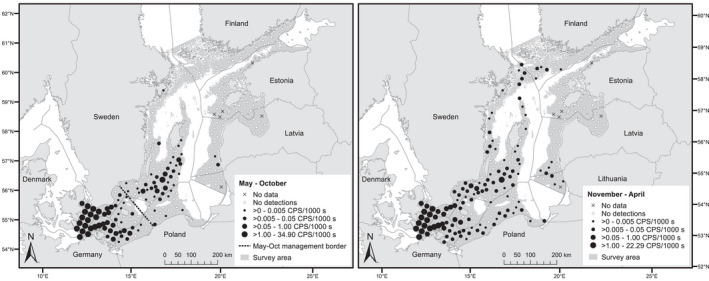
Mean acoustic detection rate of harbour porpoises during May–October and November–April. The detection rate is measured in click‐positive seconds (CPSs) per 1000 s of survey effort. The shading shows the main survey area. The May–October management border was proposed by Carlén et al. (2018)

**FIGURE A4.1:**
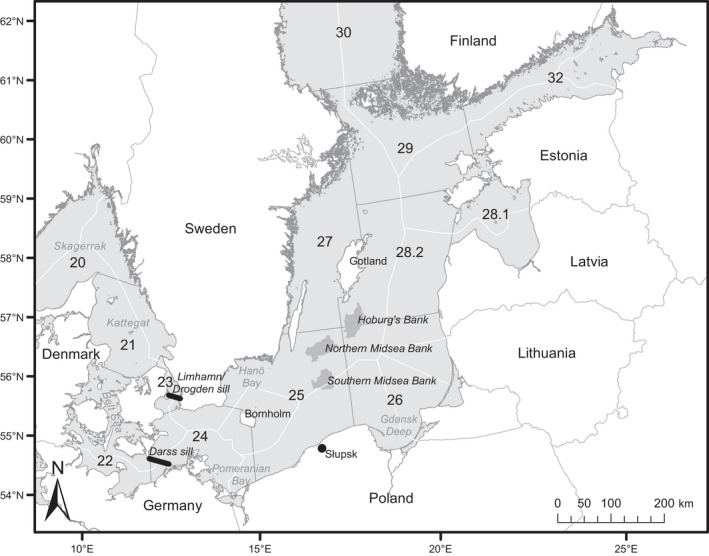
Geographical terms used in the article. The numbers indicate ICES areas. The Sound is ICES area 23. The locations of the offshore banks Hoburg's Bank, Northern Midsea Bank and Southern Midsea bank are outlined by their 30 m isobars

**FIGURE A4.2:**
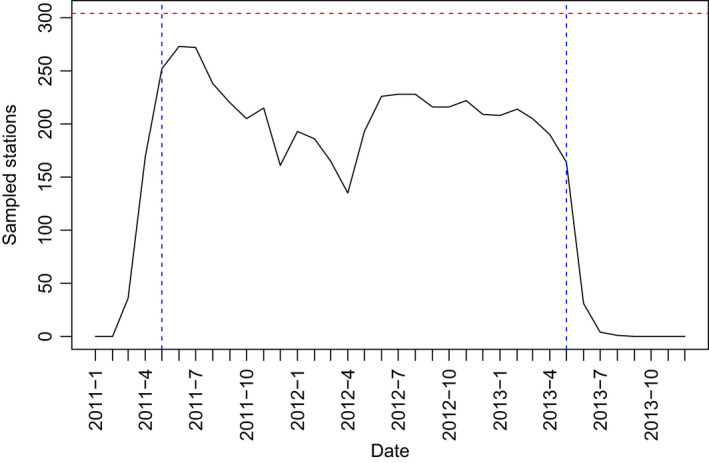
Number of sampled stations by month. The dashed blue vertical lines mark the survey period from May 1, 2011 to April 30, 2013. The dotted red horizontal line marks the total number of acoustic survey stations (304)

**FIGURE A4.3:**
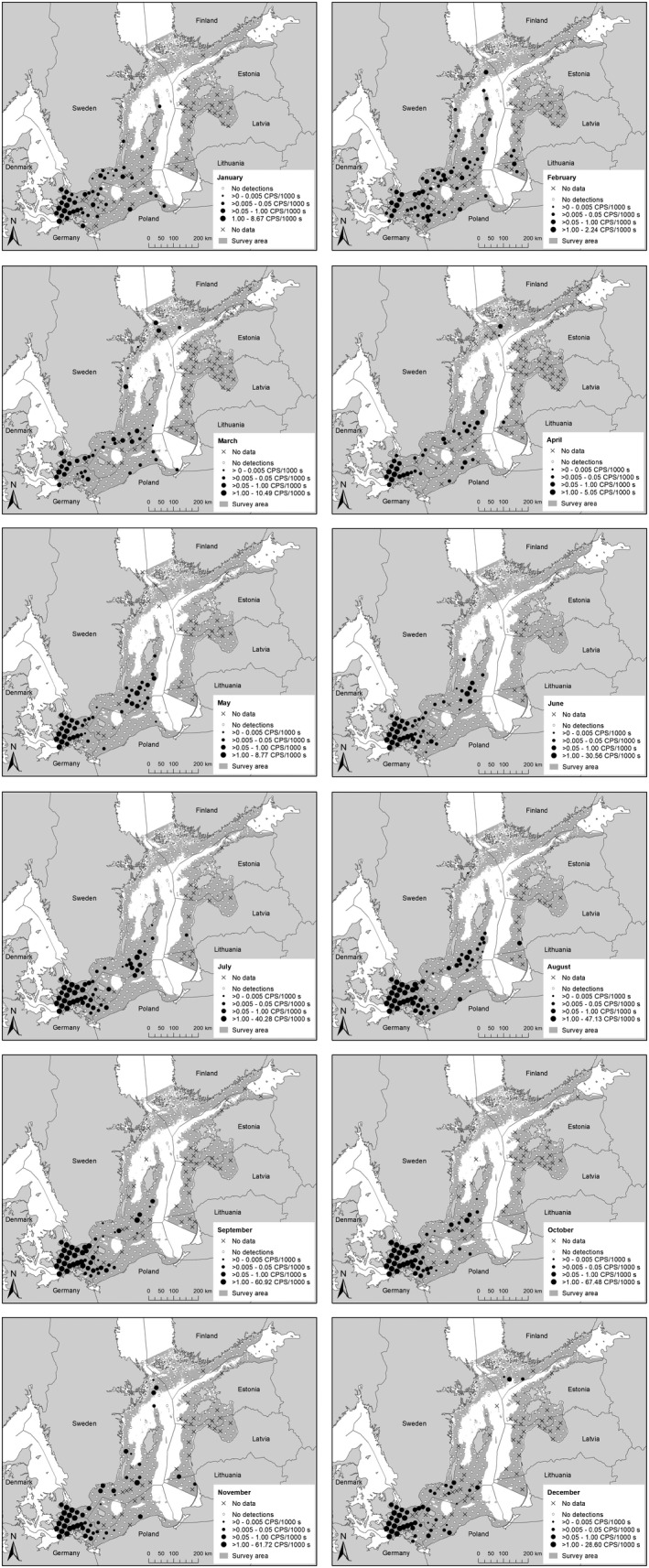
Mean acoustic detection rate of harbour porpoises by survey station and month. The detection rate is measured in click‐positive seconds (CPSs) per 1000 s of survey effort. The shading shows the main survey area

The mean acoustic detection rate (CPS per 1000 s of survey effort) from May 1, 2011, to April 30, 2013, showed a strong spatiotemporal pattern (Figure 4, Figure A4.3). During May–October, the highest mean detection rates (>1 CPS/1000 s) were recorded at the westernmost stations in Danish, Swedish, and German waters and at one station at the Northern Midsea Bank in the Baltic Proper (for geographical terms, see Figure A4.1). The second highest mean rates ([>0.05]‐1 CPS/1000 s) were recorded at the adjacent stations in the southern Swedish waters, most of the remaining stations in German waters, and two stations in western Polish waters. These rates were also recorded at five stations at and around Hoburg's and the Midsea Banks in the Baltic Proper. With few exceptions, the remaining stations with detections were adjacent to these two clusters. There were no or few detections in Finnish, Estonian, Latvian, and Lithuanian waters. During November–April, the highest mean detection rates (>1 CPS/1000 s) were again recorded in the southwest and at the same station at the Northern Midsea Bank. However, detections were made at a higher number of stations at lower rates (primarily ≤0.05 CPS/1000 s), including along the east coast of Sweden, in Finnish, Latvian, and Lithuanian waters, and along the coast of Poland. Detections were made in all countries surveyed except Estonia. Note that Russian waters were not included in this study for administrative reasons.

### 3.3 Estimation of effective detection area (EDA)

#### 3.3.1 Tracking experiment

A total of 36 tracking events took place, where for each a free‐ranging single harbour porpoise was tracked acoustically with the hydrophone array in Great Belt, Denmark, and simultaneously monitored by the adjacent 16 C‐PODs. The median track duration was 56 s (mean 64 s, range 5–263 s). Summing across all C‐PODs and tracking events, there was a total of 26,207 s of monitoring effort, of which only 137 s (0.52%) contained harbour porpoise detections on C‐PODs.

**FIGURE 5:**
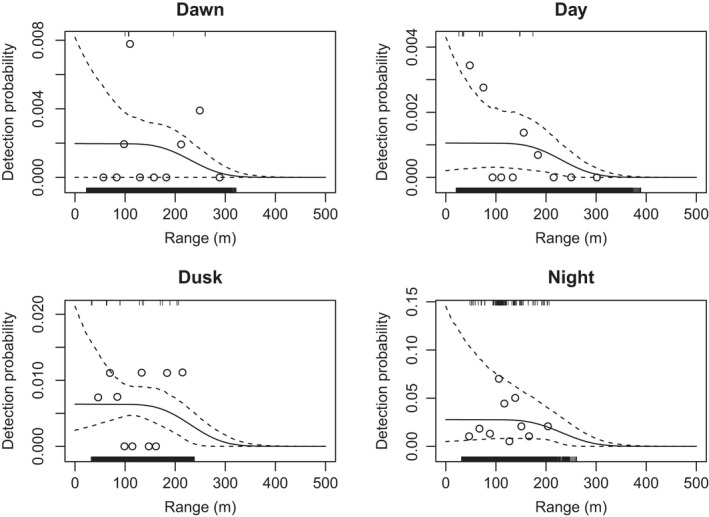
Detection function for free‐swimming porpoise from the tracking experiment. Estimated probability of detection (solid lines) and 95% bootstrap confidence limits (dashed lines) of tracked harbour porpoise in a 1‐s period in each diel phase as a function of horizontal distance. Vertical ticks at the top and bottom of each plot show the raw data: ranges at which detections were made in a 1‐s period (top of plot) or at which detections were not made (bottom of plot). Circles show a summary of these data: the proportion of positive detections in ten distance bands equally spaced through the data. The shape of the detection function (on the scale of the logit link) was constrained to be the same in all diel phases, and the function was constrained to be zero at 500 m. Note the different scales on the y‐axes

Detection probability was estimated to be approximately constant within each diel phase beyond around 150 m, declining at longer ranges; within 150 m, detection probability was estimated to be approximately 5–25 times higher at night than the other three diel phases Figure 5).

The EDA for tracked porpoises was derived from this fitted detection function and the relative acoustic detection rates in each diel phase from the main Baltic survey. Estimated EDA using just the detection function (Equation 5) ranged from 4973 m^2^ (SE 2924) at night to 188 m^2^ (SE 76) during the day (Table 1), that is, a 26‐fold difference. However, the relative acoustic detection rates in the main survey area varied only by a factor of 2.08 between day and night (Table 1). Using this information (see Materials and Methods Equation 6 and Discussion) yielded scaled estimates of EDA for tracked porpoises by diel phase that ranged from 1,851 m^2^ (SE 829) at night to 888 m^2^ (SE 398) during the day (Table 1). The scaled EDAs are equivalent to an effective detection radius ranging from 24 m at night to 16 m in the day.

**TABLE 1:** Estimated effective detection area (EDA), proportional change in detection rate, and resulting scaled EDA

Diel phase	EDA ${\hat{\upsilon }}_{d}^{**}$ [m^2^]	Proportional change (relative to Day) in detection rate ${\hat{e}}_{d}$	Scaled EDA ${\hat{\upsilon }}_{d}^{*}$ [m^2^]
Dawn	351 (224)	1.44 (0.18)	1280 (573)
Day	188 (76)	1 (0)	888 (398)
Dusk	1,138 (252)	1.21 (0.16)	1076 (482)
Night	4973 (2924)	2.08 (0.25)	1851 (829)
Weighted mean	1101 (494)	–	1101 (494)

Estimates are for a free‐swimming harbour porpoise in a 1‐second period from the tracking experiment. Values in brackets are standard errors. Symbols used (${\hat{\upsilon }}_{d}^{**}$, ${\hat{e}}_{d}$, and ${\hat{\upsilon }}_{d}^{*})$ are defined in Equations 5 and 6, which also show how the EDAs are calculated.

#### 3.3.2 Tagging study

**FIGURE A4.4:**
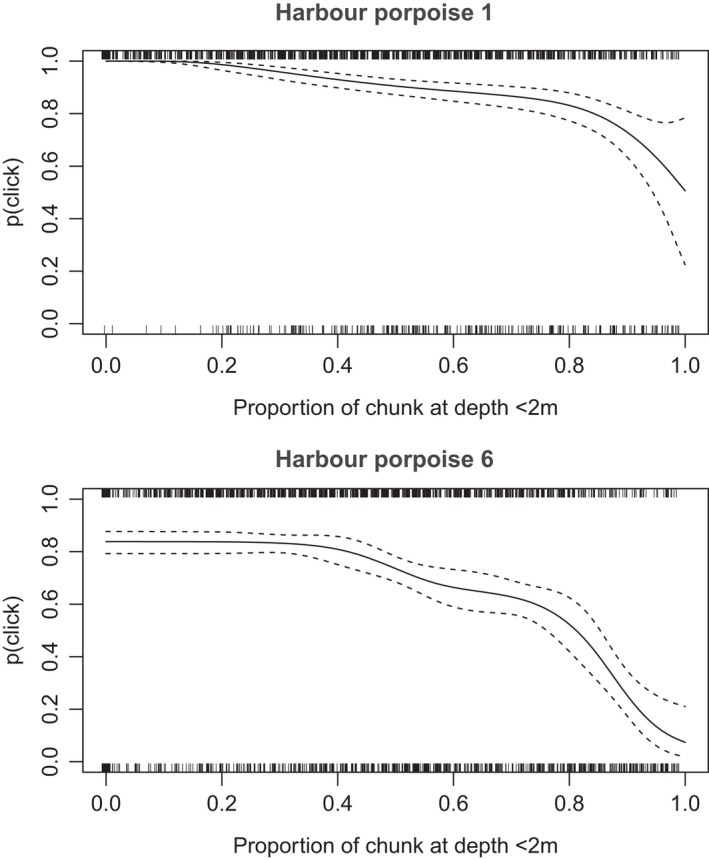
Examples of binary regression on harbour porpoise tag data to correct for missing data. Missing data were caused by truncation of data from depths <2 m. Shown here are the results for harbour porpoise 1 (top) and harbour porpoise 6 (bottom), with data collected into chunks of 54 s (the median tracking event duration). Short vertical lines at the top and bottom of each plot show the proportion of each chunk of 54 s that had depths <2 m in it (lines are horizontally jittered for clarity); lines at the top represent chunks where there was at least one porpoise click‐positive second (CPS) and lines at the bottom are chunks with no CPS. The binary regression yielded an estimate of how probability of detecting one or more CPS (p(click) on y‐axis) varied as a function of the proportion of data at depth <2 m (solid line on plot, with accompanying 95% confidence interval shows as dashed lines). We used this regression to estimate the probability of a CPS for that chunk duration if there were no missing data—in this example, this is 1.0 for harbour porpoise 1 and 0.82 for harbour porpoise 6

**FIGURE A4.5:**
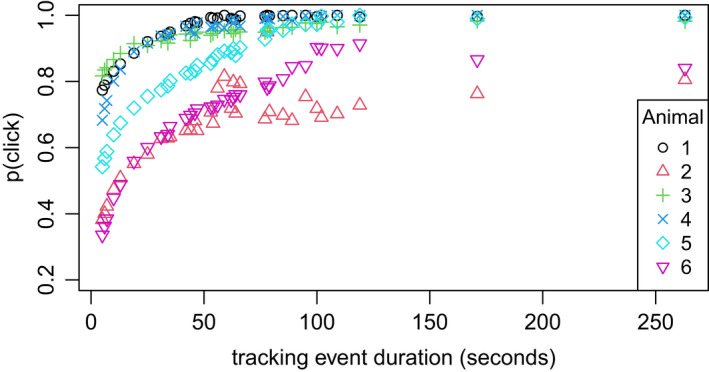
Estimated probability of one or more click‐positive seconds, p(click), for each tracking event duration and tagged harbour porpoise

Six harbour porpoises were opportunistically entrapped in Danish stationary pound nets. Duty cycled acoustic tags, recording 10 min each hour on five animals and 45 min each hour on one animal, were attached to the dorsal fins (Wright et al., 2017). Mean tag deployment duration was 5.6 days (range 2.1–11.1 days), yielding a mean of 97,362 s of recording data per animal (range 29,160–159,930 s). After truncation of data from times corresponding to when the tags were closer to the surface than 2 m (Figure A4.4), we calculated the probability of one or more CPS for each tagged animal given each tracking event duration in the tracking experiment (Figure A4.5). Averaging these probabilities across tracking event durations, the mean probability of one or more CPS varied between the six porpoises from 0.67 to 0.96 (Table A5.2). In other words, the estimated probability of a porpoise remaining silent and being missed in the tracking experiment, assuming the tagged porpoises were representative of the population sampled in the tracking experiment, ranged from 0.04 to 0.34. The average weighted probability over all animals of one or more CPS during a tracking event (denoted ${\hat{p}}_{c}$ in Materials and methods) was 0.82 (SE 0.06). A beta distribution was used to represent this uncertainty when calculating variance in abundance estimates, and the corresponding beta parameters were *a* = 37.3 and *b* = 8.1.

#### 3.3.3 Playback experiment

A total of 253 successful playback experiments of artificial porpoise click sequences were performed at 181 sampling locations within the main survey area (Table A5.3). Playbacks took place in all months of the year except January and September (Table A5.4). The number of distances per experiment at which playbacks were performed varied for operational reasons between 1 and 8, with a mean of 4; playback distances ranged from 5 to 500 m with a mean of 209 m. The general goal was to perform a playback at each survey station in each of the summer and winter seasons, but due to practical constraints with equipment failure and availability, this was not achieved.

**FIGURE A4.6:**
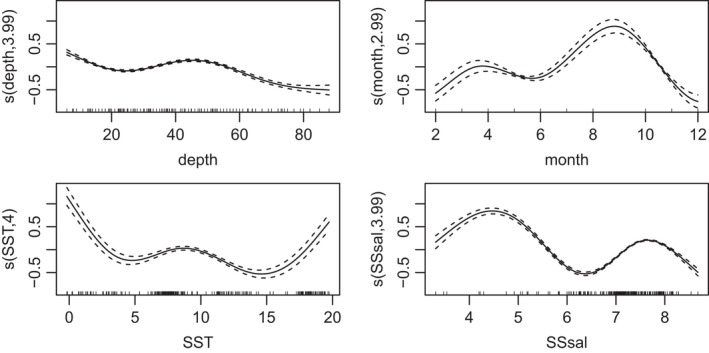
Estimated smooths in the model of playback experiments in the main study area. Solid lines show estimated smooths and dashed lines approximate 95% confidence intervals from 1‐dimensional smooth terms. Smooths are shown on the scale of the (logit) link function. Explanations for each term are given in Table A5.1. Numbers on the y‐axis after the term name indicate the equivalent degrees of freedom estimated for the smooth (with a maximum of 4). Small vertical lines inside the x‐axis indicate the location of data values

**FIGURE A4.7:**
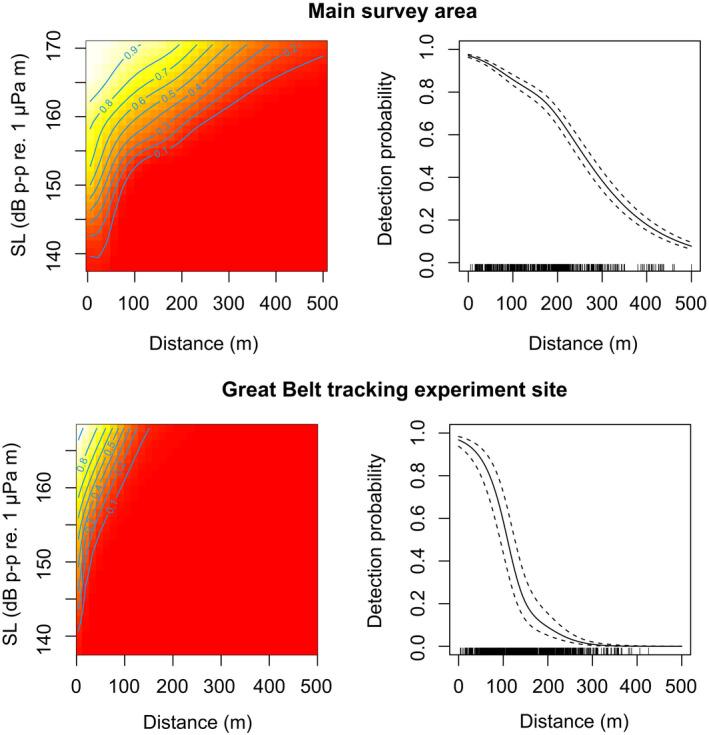
Estimated 2‐dimensional smooth for variables distance and peak‐to‐peak source level (SL_p‐p_) in the model of playback experiments. Top panel shows smooths in the main survey area and bottom panel in the Great Belt tracking experiment site. Plots are shown on the scale of the response (i.e., probability of detecting an individual click). Top plots are for sediment type (geo variable) “sand to muddy sand,” the most common type, and median values for the other variables. Bottom plots are for random effects values of zero. For both top and bottom plots, the left contour plot shows the 2‐dimensional function, with contour lines and shading indicating detection probability (red shading shows higher detection probability through to white showing lower probability). The right line plot shows the estimated effect (solid line) of distance and approximate 95% confidence interval (dashed lines) given a peak‐to‐peak source level of 168 dB re 1 µPa m (the value used to calculate effective detection area). Small vertical lines inside the x‐axis indicate the location of distances where playbacks were performed

The resulting detection/non‐detection data were used to fit the detection probability as a function of horizontal distance, source level, and other environmental factors. The selected model included a 2‐D smooth of distance and source level, plus depth, month, sea surface temperature, and sea surface salinity as continuous covariates and sediment type as a 5‐level factor (Table A5.1 and Table A5.5; Figure A4.6 and Figure A4.7 top plots). Detectability of artificial porpoise clicks decreased with distance and increased with source level (Figure A4.7 top plots). Detectability was generally lower in deeper locations, in winter months, at moderately high sea surface temperature (15°C) and higher sea surface salinity (6.5 and 8.5 PSU), although none of these relationships were monotonic (Figure A4.6).

**FIGURE A4.8:**
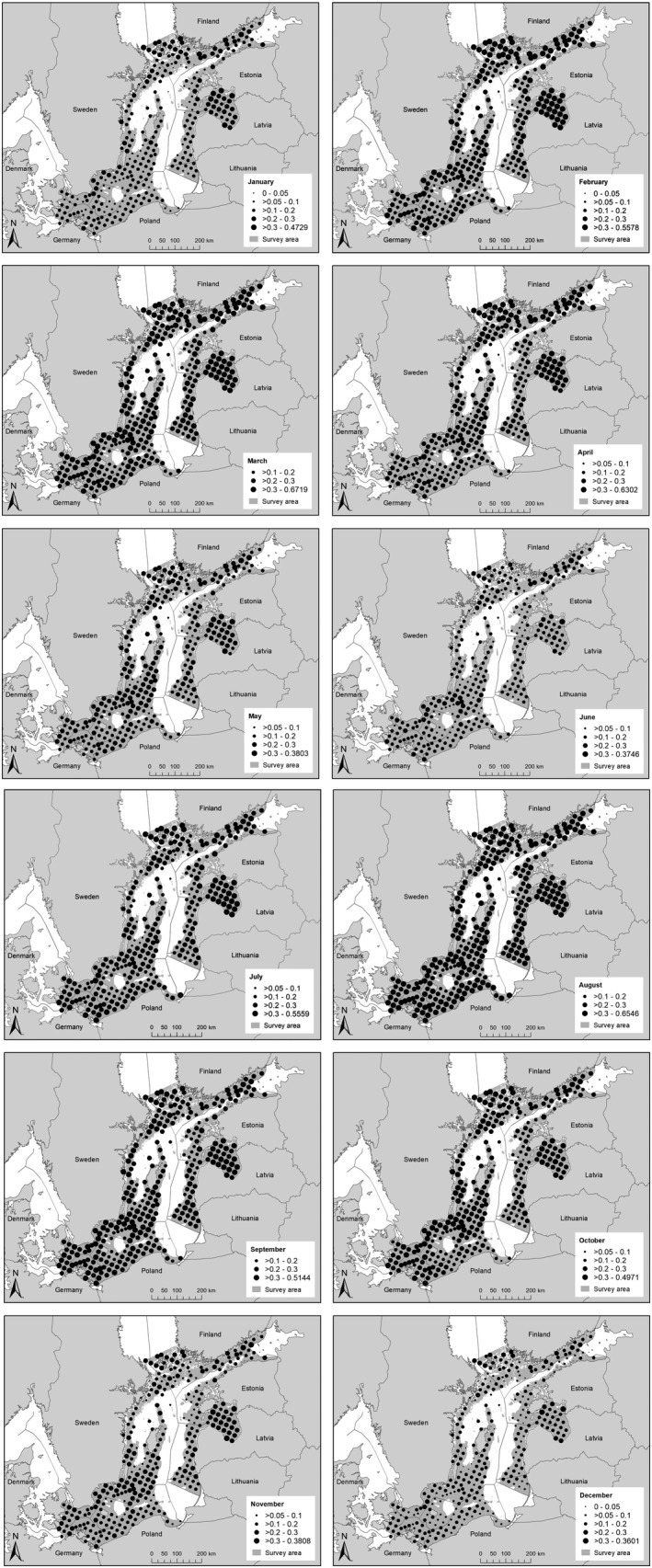
Estimated mean effective detection area (EDA, km^2^) for detection of a playback click by survey station and month

The fitted model was used to predict EDA of artificial clicks at a SL_p‐p_ of 168 dB re 1 µPa m for each sampling location and month in the main survey area. The mean EDA over all stations and months was 0.219 km^2^ (SE 0.0291), but there was considerable variation among sites and months, ranging from 0.034 km^2^ (SE 0.031, station #1097 (Sweden) in December) to 0.742 km^2^ (SE 0.213, station #3026 (Estonia) in August). In general, EDA was highest in March and August and lowest in December/January and June; it tended to be higher in the northeastern sites and lower in the more western sites (Figure A4.8).

During the tracking experiment in the Great Belt, playbacks were performed on 7 days over the study period, with 85 playbacks generated at distances ranging from 4 to 426 m (mean 155 m). Note that, unlike the main study playbacks, multiple C‐PODs were exposed to each playbacks. Again, the detection probability was modeled as a function of horizontal distance and source level, with C‐POD identifier and playbacks included as random effects (see MATERIALS AND METHODS for justification). As with the main survey, detectability of artificial porpoise clicks decreased with increasing horizontal distance and increased with increasing source level (Figure A4.7 bottom plots); however, overall detection probability was lower than for most sites in the main survey area: Estimated EDA (denoted ${\hat{\xi }}^{*}$ in the Methods) was 0.062 km^2^ (SE 0.009).

### 3.4 Density and abundance

The above elements were combined to yield estimates of density and abundance of harbour porpoise, with associated variance, by region and season (Table 2). We detected two higher‐density clusters during May–October, separated by the proposed management border (Figure 4, Carlén et al., 2018). One cluster was centered on and around the offshore banks in the central and southeastern Baltic Sea, south and southwest of the island of Gotland, Sweden (for geographical terms, see Figure A4.1). Given their distribution during the breeding season, these animals most likely belonged to the Baltic Proper population, and their total abundance in this northeast region was estimated to be 71–1,105 individuals (95% CI, point estimate 491; Table 2). Using the 20^th^ lower percentile as a precautionary minimum abundance estimate (Wade, 1998), this was equal to 138 individuals (all age classes). Assuming 50% mature individuals (Taylor et al., 2007), the mature group was estimated to be 36–553 individuals, with a 20th lower percentile of 69 individuals. The other cluster was located in the southwestern survey area, west of the island of Bornholm, Denmark, with an increasing density toward the west. Given their distribution, these animals most likely belonged to the Belt Sea population, and their abundance was estimated to be 12,350–38,849 individuals (95% CI, point estimate 21,136; Table 2). Estimates of density and abundance at the level of country, region, and season are given in Table A5.6 and Table A5.7.

**TABLE 2:** Estimates of density and abundance of harbour porpoises in the Baltic Sea survey area (northeast and southwest of the May–October management border as well as total area) during May–October and November–April

Region	Season	Area (km^2^)	Density (animals/1000 km^2^)		Abundance		CV (%)
			Estimate	95% CI	Estimate	95% CI	
Northeast	May–Oct	132,603	3.70	0.54–8.33	491	71–1105	68.0
Northeast	Nov–April	132,603	1.83	0.71–4.22	243	94–560	54.1
Southwest	May–Oct	33,982	621.98	363.43–1143.21	21,136	12,350–38,849	33.4
Southwest	Nov–April	33,982	316.05	155.24–702.10	10,740	5275–23,859	45.3
Total	May–Oct	166,585	129.83	77.66–239.02	21,627	12,937–39,816	33.0
Total	Nov–April	166,585	65.93	33.17–147.35	10,983	5525–24,546	44.8

Note

CI, confidence interval; CV, coefficient of variation.

The distribution was more scattered during November–April, but still with the highest density in the southwest, albeit lower than during May–October, and still with a considerable number of harbour porpoises on the offshore banks in central Baltic Proper (Figure 4). In the entire surveyed area during November–April, the total abundance was estimated to be 5,525–24,546 animals (95% CI, point estimate 10,983; Table 2). During November–April, the number of porpoises remaining northeast of the May–October management border in Figure 2 was estimated to be 94–560 (95% CI, point estimate 243), and southwest of this line, 5,275–23,859 animals (95% CI, point estimate 10,740). The wide confidence intervals of the abundance estimates mean that the November–April estimates were not statistically different from the May–October estimates (bootstrap 95% CIs on the difference between winter and summer estimates include zero for the northeast (−812 to 317) and southwest (−27,160 to 3,874) regions).

## 4 DISCUSSION

### 4.1 Abundance estimates

#### 4.1.1 Separate populations (May–October)

We successfully estimated the density and abundance of a rare odontocete population. During May–October, that is, during the breeding season, 71–1,105 harbour porpoises (95% CI, point estimate 491) were identified in the northeast region of the main survey area, northeast of the proposed management border shown in Figure 2. We believe these represent the main part of the Critically Endangered (CR) Baltic Proper population. The animals were centered on and around the shallow offshore banks south and southwest of the Island of Gotland, Sweden (Carlén et al., 2018). Prior studies on genetics, morphology, acoustics, and movement (Galatius et al., 2012; Lah et al., 2016; Sveegaard et al., 2015; Wiemann et al., 2010) support the assumption that this cluster represents the “true” Baltic Proper population. At the same time, 12,350–38,849 harbour porpoises (95% CI, point estimate 21,136) were found in the southwest region of the main survey area, primarily west of the island of Bornholm, Denmark. We believe that the main part of these animals belong to the Belt Sea population, which is centered in the Belt Sea (Carlén et al., 2018; Sveegaard et al., 2015). The estimated density in this region was 0.36–1.14 animals per km^2^ (95% CI, point estimate 0.62). Visual surveys have been carried with partial overlap with the southwest region. The latest visual surveys covering the major part of the Belt Sea population in July 2012 (Viquerat et al., 2014) and 2016 (Hammond et al., 2017) estimated densities of 0.50–1.24 animals per km^2^ (95% CI, point estimate 0.79) and 0.58–1.85 (95% CI, calculated by us from CV = 0.30 and point estimate 1.04 assuming a log‐normal distribution). Further, eight German surveys have been carried out during May–October 2002–2006, with 32% overlap with the southwest region (stratum G, Scheidat et al., 2008). During four of these visual surveys, no harbour porpoise were observed in the overlapping area. For the remaining four surveys, the density was estimated to 0.06–3.19, 0.00–0.03, 0.00–0.20, and 0.00–0.02 animals per km^2^ (95% CI, point estimates 0.004, 0.008, 0.058 and 1.016). Due to the limited overlap in time and space, and the fact that the visual surveys represents days and the acoustic monitoring years, the results cannot be directly compared. However, since the distribution pattern of Belt Sea porpoises equipped with satellite transmitters shows a sharp decrease from the Belt Sea toward Bornholm (Mikkelsen et al., 2016; Sveegaard et al., 2015), the true density in the southwest region of the main survey area is more likely to be in the lower than the upper end of our confidence interval.

#### 4.1.2 Mixed populations (November–April)

During November–April, the harbour porpoises were more dispersed and showed no clear spatial separation between the Baltic Proper and Belt Sea populations (Carlén et al., 2018). Even though the overall detection rates decreased, there was still a relatively high detection rate of porpoises on the shallow banks in the central Baltic Proper, and the detection rates increased along the Polish coast as well as in Hanö Bay, Sweden, on both sides of the May–October management border in Figure 2 (Figure A4.1). The number of animals remaining northeast of the May–October management border was 94–560 porpoises (95% CI, point estimate 243), around half the estimated number during May–October, but the wide confidence intervals in both periods mean these values are not statistically different. Earlier studies have shown movements of porpoises into the German Pomeranian Bay during winter, proposed to be Baltic Proper animals (Benke et al., 2014; Gallus et al., 2012). Our results neither confirm nor reject this hypothesis, yet it seems likely that there is a net migration of Baltic Proper porpoises from the northeast to the southwest region during November–April. This movement would imply that conservation measures for the Baltic Proper porpoise population, such as bycatch mitigation, should cover the waters from the southwestern Baltic Sea to the Åland and Archipelago Seas during November–April (ICES, 2020a). Management measures that only cover the offshore banks and surrounding areas during the summer months would not be adequate to protect the population.

Even though Baltic Proper animals move into the southwest region during November–April, the majority of the animals in this region still belongs to the more abundant Belt Sea population. During these months, the abundance in the southwest region decreased to 5,275–23,859 individuals (95% CI, point estimate 10,740). Although this number is considerably lower than the May–October estimate, it is not statistically different due to the wide confidence intervals. Nevertheless, such a seasonal migration pattern is consistent with earlier studies (Benke et al., 2014; Gallus et al., 2012; Sveegaard et al., 2015; Verfuβ et al., 2007) that found movement of Belt Sea harbour porpoises from the southwest region to the northwest, into the Belt Sea, during the winter.

### 4.2 Conservation status, threats, and management needs

IUCN and HELCOM have classified the harbour porpoises in the Baltic Proper as Critically Endangered (CR; Hammond et al., 2008; HELCOM, 2013). The assessments were based on an aerial survey in 1995, partially covering the currently known management range of the Belt Sea population and partially the currently known Baltic Proper management range (Carlén et al., 2018; Sveegaard et al., 2015). The aerial survey estimated a total of 599 groups of single animals (95% CI 200–3,300 groups) (Hiby & Lovell, 1996). Based on an estimation of 50% mature individuals (Taylor et al., 2007), and a precautionary approach using the lower 20^th^ percentile of the abundance estimate (Wade, 1998), IUCN reached an estimate of 192 mature individuals. We have now estimated the population abundance of the Baltic Proper population to be 71–1,105 individuals, with a 20th lower percentile equal to 138 (all age classes). Assuming 50% mature individuals, 36–553 mature Baltic Proper harbour porpoises remain with a 20th lower percentile of 69. These low numbers strongly support the IUCN and HELCOM assessment that the Baltic Proper harbour porpoise is facing an extremely high risk of extinction in the wild.

In its latest threat matrix for the Baltic Proper harbour porpoise, ICES Working Group on Marine Mammal Ecology (WGMME) lists the threat levels by bycatch, contaminants, and underwater noise from explosions, military sonars, and seismic surveys as “high,” based on evidence or strong likelihood of negative population effects, mediated through effects on individual mortality, health, and/or reproduction (ICES, 2019). For the years 2009–2012, the annual number of bycaught harbour porpoises of the Baltic Proper population has been estimated to 7–12 animals (North Atlantic Marine Mammal Commission & Norwegian Institute of Marine Research, 2019). This is ten times or more than the estimated limit for sustainable human‐caused mortality for the population: 0.7 animals per year (North Atlantic Marine Mammal Commission & Norwegian Institute of Marine Research, 2019), using the PBR (Potential Biological Removal) approach (Wade, 1998). In the Baltic Proper, 97% or more of harbour porpoise bycatch have been reported to occur in gillnets, including driftnets (prior to 2008) and semi‐driftnets (Berggren, 1994; EC‐DGMARE, 2014; Skóra & Kuklik, 2003). As pingers reduce but do not eliminate bycatch of harbour porpoises (Dawson et al., 2013; Larsen & Eigaard, 2014; Palka et al., 2008), a bycatch rate close to zero can only be reached by closing all gillnet fisheries within the distribution range of the Baltic Proper harbour porpoise (ICES, 2020a).

Polychlorinated biphenyls have been associated with impaired health, immunosuppression, increased disease risk, and reproductive failure in harbour porpoises (Beineke et al., 2005, 2007, 2007; Jepson et al., 1999, 2005; Lehnert et al., 2019; Murphy et al., 2015). PCB concentrations measured in harbour porpoises collected the Baltic Sea in the 1980s and 1990s have been alarmingly high (Berggren et al., 1999; Bruhn et al., 1999; Falandysz et al., 2002; Kannan et al., 1993). The recorded levels were often well above thresholds for the onset of physiological impacts, adverse health effects, and profound reproductive impairment (Helle et al., 1976; Jepson et al., 2005; Kannan et al., 2000; Murphy et al., 2015). Since the 1990s, the PCB concentrations in Baltic herring (*Clupea harengus*) and guillemot egg (*Uria aalge*) have declined, but remain higher than, for example, in the North Sea (Nyberg et al., 2015). The current levels in the Baltic biota indicate that PCB contamination remains a serious impediment to the health and reproductive status of the Baltic Proper harbour porpoise population, but lack of samples prevents direct studies. The lack of samples is due to a combination of the small population size and a low willingness to report and land bycaught harbour porpoises.

Impulsive underwater noise sources occurring in the Baltic Proper can cause behavioral disturbance, hearing loss, and other physical injury to harbour porpoises (Kastelein et al., 2015, 2017; Ketten, 2004; Lucke et al., 2009; Pirotta et al., 2014; Sarnocińska et al., 2020; Thompson et al., 2013; von Benda‐Beckmann et al., 2015). Data on loud sources of impulsive noise in the Baltic Sea are collated nationally and reported to an ICES registry in support of HELCOM (HELCOM, 2021; ICES, 2020b). During 2015–2019, underwater explosions have primarily been reported from a few and primarily coastal locations in the Baltic Proper, airgun arrays in offshore waters in the southern Baltic Proper, and sonars in offshore waters across the Baltic Proper (ICES, 2020b). The spatial distribution of the sonars, which primarily are used for sea floor exploration, strongly overlaps with the year‐round distribution of Baltic Proper harbour porpoise. The pressure is rapidly increasing due to a raising interest in offshore wind power. In January 2020, the total number of wind farms in the stages from concept to pre‐construction within the entire main survey area was 58, whereof 39 are within the May–October management range of the Baltic Proper population (4COffshore, 2020; Table A5.8). It is therefore concerning that there is a lack of regulations regarding underwater noise. Germany has a dual exposure limit to avoid injury and significant disturbance from pile driving, applicable only to harbour porpoises in the southern North Sea (Federal Ministry for the Environment, Nature Conservation & Nuclear Safety, 2013), while Denmark has an exposure limit to avoid hearing impairment from pile driving, together with a guideline for estimating such impact, applicable to any Danish waters (Danish Energy Agency, 2016; Skjellerup et al., 2015). In all other countries around the Baltic Sea, underwater noise exposure limits are missing, and no country has any noise guidelines that take the conservation status of the Baltic Proper harbour porpoise into account. This is despite the fact that underwater noise is listed as a pollutant in the European Marine Strategy Framework Directive (2008/56/EC), and offshore constructions and associated activities pose a high risk to negatively impact the status of the Critically Endangered Baltic Proper harbour porpoise population. However, the development of common standards for impact assessment and mitigation of impulsive noise is a prioritized action in the HELCOM draft regional action plan for underwater noise (HELCOM 2021).

A recent population viability assessment of the Baltic Proper harbour porpoise population has been carried out, applying a range of biologically realistic parameter values and three different levels of bycatch (Cervin et al., 2020). Under the baseline scenario, with biological values representing a healthy population and absence of bycatch, the annual population growth rate was estimated to 2.3% (SD ±6.4%). Under recent conditions, a more likely scenario is an intermediate fertility (60%) in combination with a bycatch of 7–15 individuals per year (7–12 bycatch per year was estimated for 2009–2012 by North Atlantic Marine Mammal Commission & Norwegian Institute of Marine Research, 2019). The latter scenario was estimated to lead to quasi‐extinction (≤50 animals) in 44–75 years. Even substantial improvements in fertility could not balance out the investigated levels of bycatch (Cervin et al., 2020).

The importance of adequate bycatch mitigation on the population development is clearly demonstrated by the examples of the vaquita (*Phocoena sinus*), a porpoise species endemic to the Gulf of California, Mexico, and the Morro Bay harbour porpoise stock in Central California, USA. The abundance estimates of both management units have been similar to our estimate of the Baltic Proper harbour porpoise, and both units have been threatened by bycatch, but differences in the efficiency of the bycatch mitigation have led to strikingly different outcomes. In 1997, the abundance of the vaquita was estimated to be 567 individuals (95% CI 177–1073). Despite several efforts (Jaramillo‐Legorreta et al., 2017; Rojas‐Bracho & Reeves, 2013), bycatch in illegal gillnetting has continued (Jaramillo‐Legorreta et al., 2017, 2019), resulting in fewer than 19 vaquitas remaining as of summer 2018 (Jaramillo‐Legorreta et al., 2019) with extinction becoming increasingly probable without immediate elimination of all bycatch. In contrast, high levels of bycatch in set gillnets within the range of the Morro Bay harbour porpoise stock lead to increasingly restrictive closures, reaching an almost complete ban (Forney et al., 2014; Moore et al., 2009). Additional bycatch in a driftnet fishery was reduced by the use of acoustic deterrent devices (pingers) and closures (Barlow & Cameron, 2003; Moore et al., 2009). From 1990 to 2012, the Morro Bay stock increased from 571 (95% credible interval 252–2666) to 4191 animals (95% credible interval 1900–11,971), indicating an average annual growth rate of 9.6% since the near elimination of gillnets (Forney et al., 2020). It should be pointed out that the Morro Bay harbour porpoise stock does not suffer from high levels of environmental pollutants as does the Baltic Proper harbour porpoise population.

These two examples show that a severely reduced porpoise population may recover if the human‐induced mortality is considerably reduced, while failing to implement and enforce prompt and decisive conservation measures, often requiring community acceptance, may lead to extinction. They also show that repeated abundance surveys provide a thorough basis for informed measures. However, a major difference between the Baltic Proper harbour porpoise, the vaquita and the Morro Bay harbour porpoise stock, is that the distribution range of the Baltic Proper harbour porpoise is approximately 12 and 22 times larger respectively, and is shared by nine countries. As such, efficient international cooperation to conserve the Baltic Proper harbour porpoise is needed.

### 4.3 Methodological limitations and alternatives

#### 4.3.1 Main survey

As we excluded waters deeper than 80 m from the main survey area, it was not possible to quantify the number of porpoises there. Within the surveyed depth range, most harbour porpoise detections occurred at 20–50 m depth and tapered off on both sides, especially toward greater depths (Carlén et al., 2018). There is no information on association between harbour porpoise and fish distribution in the central Baltic Sea. However, prey availability and predictability appear to be the main driver for harbour porpoise distribution in The Sound, the strait that forms the Danish–Swedish border (Sveegaard, Andreasen, et al., 2012), and herring distribution explains large‐scale distribution of harbour porpoises in the eastern North Sea, Skagerrak, and Kattegat (Sveegaard, Nielsen, et al., 2012). In the southern central Baltic Sea, the most abundant subgroup of herring spawns in shallow coastal areas in spring. This behavior is, in general, followed by a migration by older herring to the deep offshore Bornholm Basin and Gdansk Deep from July to December. Sprat (*Sprattus sprattus*) perform the opposite seasonal migration; they concentrate in the Bornholm Basin, Gdansk Deep, and Gotland Basin from December to June and transit to shallow coastal waters from June to December (Aro, 2002; Parmanne et al., 1994; Popiel, 1984; Stepputtis, 2006). Pelagic prey are thus available for harbour porpoises in both shallow and deep Baltic waters year‐round, while benthic prey are only available in shallow waters due to anoxic conditions (Hansson & Andersson, 2015). Regardless, future surveys are recommended to investigate the occurrence of harbour porpoises in the deep waters of the Baltic Sea.

We assumed that porpoise density at the sampled locations was, on average, representative of that in the main survey area. This was ensured by the systematic random grid design, although some adjustments had to be made in the few cases where the primary grid location could not be surveyed (Carlén et al., 2018). Overall, we believe these deviations from the ideal design will have caused a negligible bias in the abundance estimate. For stations that were surveyed, there was geographic variation in coverage (again for logistical reasons), with lower coverage in the east of the main survey area. While this lower coverage was accounted for in the analysis methods, and so will not cause bias, it does mean that uncertainty is higher in this region. One assumption made in dealing with missing data is that, within station and month, it is missing at random with respect to animal density.

In using the detection metric of click‐positive second (CPS) as being proportional to porpoise density (Equation 1 in Materials and methods), we assumed that at most one porpoise was detected in a one‐second snapshot at a sampling station. This assumption is justified because of the highly directional nature of porpoise click production: even when larger groups of porpoises are present, it is unlikely that more than one will be facing a hydrophone in the same second. Various alternative metrics have been used in passive acoustic monitoring with C‐PODs and the preceding T‐PODs, such as the number of detected clicks per unit time (Jaramillo‐Legorreta et al., 2019; Osiecka et al., 2020), encounter rate and duration (Benjamins et al., 2016; Carlström, 2005), and detection positive time units ranging from 15 s or one minute (Clay et al., 2018; Kyhn et al., 2012; Nuuttila et al., 2018), to hours (Benjamins et al., 2017), waiting times or silent periods (Carstensen et al., 2006; Dähne, Gilles, et al., 2013) or days (Benke et al., 2014; Palmer et al., 2019). Click counting is an example of a cue‐based approach that has been recognized as a valid method for estimating absolute density (e.g., Marques et al., 2013). However, the porpoise detection algorithm used here (and generally for C‐PODs) requires multiple clicks to be received, and although decreasing the risk of false positives, it complicates the process of estimating click detectability and linking it to click production rate. The number of clicks received per unit time (e.g., per second) given that at least one is detected is also highly variable, partly because click production rate varies considerably with behavior and click type (buzz clicks, e.g., are produced with a much shorter inter‐click interval). Given this variability, an approach based on using acoustics to detect animal presence at “snapshots” of time was deemed preferable for this study. Using a short snapshot interval enabled us to assume that at most one animal was detected per snapshot and so bypass the need to estimate population mean group size; robust estimates of group size are not available for harbour porpoises in the Baltic Sea (Berggren et al., 2002). In addition, longer “porpoise positive” time units such as hours or days will saturate at higher density so they become no longer proportional to animal density.

The estimation method assumed no false‐positive CPSs. This assumption was supported by a detailed manual analysis that showed negligible false‐positive detections from the classification algorithm used (see Appendix 2). The disadvantage of using such a stringent algorithm is that a large number of valid detections are discarded, due to a restrictive classification criterion, contributing to an effective detection area that was much smaller than the area over which it is possible to detect porpoise clicks. Because only a small area was monitored around each station, the detection rate variance was high. False‐positive detections are not a problem for abundance estimation, as long as their rate is accurately determined (Marques et al., 2013). In the current case, there was a strong impetus to minimize false detections in order to avoid incorrectly claiming the presence of the species based on false‐positive detections, since this would have substantial implications for the conservation obligations of the countries around the Baltic Sea. In other applications, a more liberal classification algorithm would be preferred and would lead to a lower overall variance.

#### 4.3.2 Tracking experiment, tagging study, and playback experiment

Our estimates of effective detection area per station and month were based on the tracking experiment in the Great Belt, the tagging study and the playback experiment (Equation 3 in Materials and methods). In the tracking experiment, we assumed that only one animal was present during each tracking event; we excluded data from times where we could visually detect multiple animals or saw evidence of multiple animals in the acoustic tracking data. We assumed that the animals were accurately localized by the acoustic tracking array; in practice, there will have been some localization error but its effect on inference is likely minimal. We assumed the acoustic behavior of porpoises tracked in the Great Belt site was representative of that in the main survey area—an assumption that is unlikely to be correct. Indeed, we found that the estimation of variation in detectability with diel phase in the Great Belt tracking experiment was far greater than the diel variation in acoustic detection rate from the main survey. This diel variation could be, for example, because porpoises were foraging on prey that is more accessible at night during the tracking experiment and so were more vocally active in that diel phase compared with other places within the main survey area. Other possible explanations may be differences in the vertical migratory behavior of fish, affecting the vertical distribution and/or orientation of porpoises. Alternatively, there may be diel differences in click propagation or masking noise, although it is hard to come up with a plausible mechanism for these. Prompted by suggestions from the reviewers, we undertook an examination of whether the tag or playback data showed any diel patterns (Appendix 3). We found no consistent diel pattern across tagged porpoises in either vertical distribution (crudely summarized as proportion of time below 2 m depth) or proportion of CPSs. We did find a small increase in detectability of playback clicks at night across the main survey area, but there was a small decrease in detectability at dusk and dawn which is not consistent with the observed patterns in click detections in the main survey. Hence, the results of this additional examination were inconclusive and point to the need for further research. Although in our analysis we corrected for diel variation in detection rates, our reliance on one site for estimating detectability of wild‐swimming porpoises is probably the biggest weakness of our study. Future abundance estimation surveys should collect such information from a larger sample of sites, and within the survey area, to increase robustness of the estimates. Our tracking experiment also had a small sample size of independent tracking events, which did not cause bias, but contributed greatly to overall variance. Future studies should devote a bigger proportion of the overall effort to collecting detectability data from animal encounters, which will likely necessitate using lower cost detectability measurement methods than the tracking experiment. A suitable method would be multiple deployments of vertical hydrophone arrays with four or more channels, allowing distances to be calculated up to approximately 70–100 m (Dähne et al., 2020; Kyhn et al., 2013). However, to gather sufficient click data in the Baltic Proper, these systems would have to work autonomously over long time frames (at least weeks to months).

Data from tagged animals were used to account for the small proportion of animals that could have been missed from the tracking experiment because they did not emit echolocation clicks while in the vicinity of the tracking array. We assumed that the acoustic behavior of the tagged animals was representative of those in the Great Belt. This is not something we can test directly, but we did find a relatively small variation between the six tagged animals in the mean probability of one or more CPS in a time period corresponding with the length of the tracking events (Table A5.2). This small variation indicates that the average acoustic behavior at this time scale may not vary greatly between individuals. The relatively small variation also meant that, despite the small sample of only six tagged individuals, the estimate of mean probability of a CPS had low variance and contributed little to overall uncertainty in abundance estimates. The tags do not effectively record clicks while they are close to the surface, and hence, we also had to assume that click production while animals were close to the surface was the same as that while they were deeper. While it may be the case that click production is less at shallow depths (certainly no clicks can be recorded while the animal is above the surface to breathe), the periods of time at these depths are generally much shorter than the length of the tracking events, and so mild violation of this assumption is unlikely to cause much bias in the results.

One possible factor affecting porpoise acoustic behavior is group size. The tracking experiment included only lone individuals, and hence, if acoustic behavior while echolocating is a function of group size, then this could potentially bias estimates of EDA derived from this experiment. If, on the other hand, group size affects the probability that a porpoise within the group echolocates at all over a longer period, then this would be part of the tagging study estimation of probability of clicking. Bias could arise here if probability of echolocation depends on group size and group size varies substantially over the main survey area or by month.

We used playbacks of artificial porpoise clicks to determine how the effective detection area calculated from wild‐swimming porpoises in the tracking experiment scaled to each sampling location in the main survey area, and how the scaling changed by month. Compared with observations on wild‐swimming porpoises, playback experiments are easy to perform. A hardware failure meant we obtained fewer playbacks than expected, and in some places, a larger range of distances from the C‐PODs would have been helpful, but overall the estimated detection functions were robust and had low variance. Playback experiments are an excellent way to estimate the effects of variation in sensor depth and changing propagation conditions, but because they do not include porpoise behavior or (in our case) the directionality of porpoise clicks, they are no substitute for observations of wild‐swimming animals. However, given the extremely low porpoise density in most parts of the Baltic Sea, it will never be possible to estimate detectability using wild‐swimming porpoises in all areas, and hence, some component of playback‐measured calibration will be necessary also in future studies.

## 5 CONCLUSIONS

An international effort of eight European countries reliably estimated the abundance of a rare and cryptic animal population across a large spatial scale using passive acoustic monitoring. We obtained a small abundance estimate for the Baltic Proper harbour porpoise, confirming that the population is facing an extremely high risk of extinction. Given the large geographical scale in which the population is distributed, the fact that its distribution range is shared by nine different countries, and the importance in taking action promptly, we call for immediate, urgent, and efficient international cooperation in eliminating bycatch and mitigating the negative impact of underwater noise and other environmental pollutants on harbor porpoises in the Baltic Sea.

## CONFLICT OF INTEREST

Nick Tregenza has designed, manufactures, and is the supplier of C‐PODs. No other author has any conflict of interest to declare.

### AUTHOR CONTRIBUTION

**Mats Amundin:** Conceptualization (equal); Funding acquisition (lead); Investigation (equal); Methodology (equal); Project administration (equal); Resources (equal); Validation (equal); Visualization (equal); Writing – original draft (equal); Writing – review & editing (equal). **Julia Carlström:** Conceptualization (equal); Funding acquisition (lead); Methodology (equal); Project administration (lead); Supervision (lead); Validation (equal); Visualization (equal); Writing – original draft (lead); Writing – review & editing (lead). **Len Thomas:** Conceptualization (equal); Formal analysis (lead); Methodology (lead); Visualization (equal); Writing – original draft (lead); Writing – review & editing (equal). **Ida Carlén:** Conceptualization (equal); Funding acquisition (lead); Project administration (lead); Visualization (equal); Writing – review & editing (equal). **Jens Koblitz:** Funding acquisition (equal); Investigation (equal); Methodology (equal); Project administration (equal); Writing – review & editing (equal). **Jonas Teilmann:** Conceptualization (equal); Funding acquisition (equal); Investigation (equal); Methodology (equal); Project administration (equal); Writing – review & editing (equal). **Jakob Tougaard:** Conceptualization (equal); Methodology (equal); Writing – review & editing (equal). **Nick Tregenza:** Formal analysis (equal); Methodology (equal); Resources (equal); Software (equal); Validation (equal). **Daniel Wennerberg:** Data curation (lead); Investigation (equal); Resources (equal); Software (equal); Validation (equal). **Olli Loisa:** Data curation (equal); Formal analysis (equal); Funding acquisition (equal); Investigation (equal); Methodology (equal); Project administration (equal); Validation (equal); Writing – review & editing (equal). **Katharina Brundiers:** Formal analysis (equal); Investigation (equal); Visualization (equal); Writing – review & editing (equal). **Monika Kosecka:** Formal analysis (equal); Investigation (equal). **Line A. Kyhn:** Data curation (equal); Investigation (equal); Supervision (equal); Validation (equal); Writing – review & editing (equal). **Cinthia Tiberi Ljungqvist:** Data curation (equal); Investigation (equal); Project administration (equal); Resources (equal); Software (equal); Supervision (equal); Validation (equal); Writing – review & editing (equal). **Signe Sveegaard:** Investigation (equal); Writing – review & editing (equal). **M. Louise Burt:** Formal analysis (equal); Methodology (equal); Writing – review & editing (equal). **Iwona Pawliczka:** Conceptualization (equal); Funding acquisition (equal); Investigation (equal); Project administration (equal); Resources (equal); Supervision (equal); Writing – review & editing (equal). **Ivar Jussi:** Investigation (equal); Methodology (equal). **Radomil Koza:** Data curation (equal); Investigation (equal); Validation (equal). **Bartlomiej Arciszewski:** Investigation (equal). **Anders Galatius:** Investigation (equal); Writing – review & editing (equal). **Martin Jabbusch:** Investigation (equal); Methodology (equal); Project administration (equal); Resources (equal). **Jussi Laaksonlaita:** Investigation (equal); Methodology (equal); Writing – review & editing (equal). **Sami Lyytinen:** Investigation (equal); Methodology (equal); Writing – review & editing (equal). **Jussi Niemi:** Investigation (equal); Methodology (equal); Writing – review & editing (equal). **Aleksej Šaškov:** Investigation (equal). **Jamie MacAuley:** Data curation (equal); Formal analysis (equal); Investigation (equal); Methodology (equal); Software (equal). **Andrew J. Wright:** Formal analysis (equal); Investigation (equal); Methodology (equal); Resources (equal); Software (equal); Validation (equal). **Anja Gallus:** Funding acquisition (equal); Project administration (equal). **Penina Blankett:** Conceptualization (equal); Funding acquisition (equal); Writing – review & editing (equal). **Michael Dähne:** Conceptualization (equal); Funding acquisition (equal); Writing – review & editing (equal). **Alejandro Acevedo‐Gutiérrez:** Writing – original draft (equal); Writing – review & editing (equal). **Harald Benke:** Conceptualization (equal); Funding acquisition (equal); Project administration (equal); Resources (equal); Supervision (equal).

## Figures and Tables

**FIGURE A3.1:**
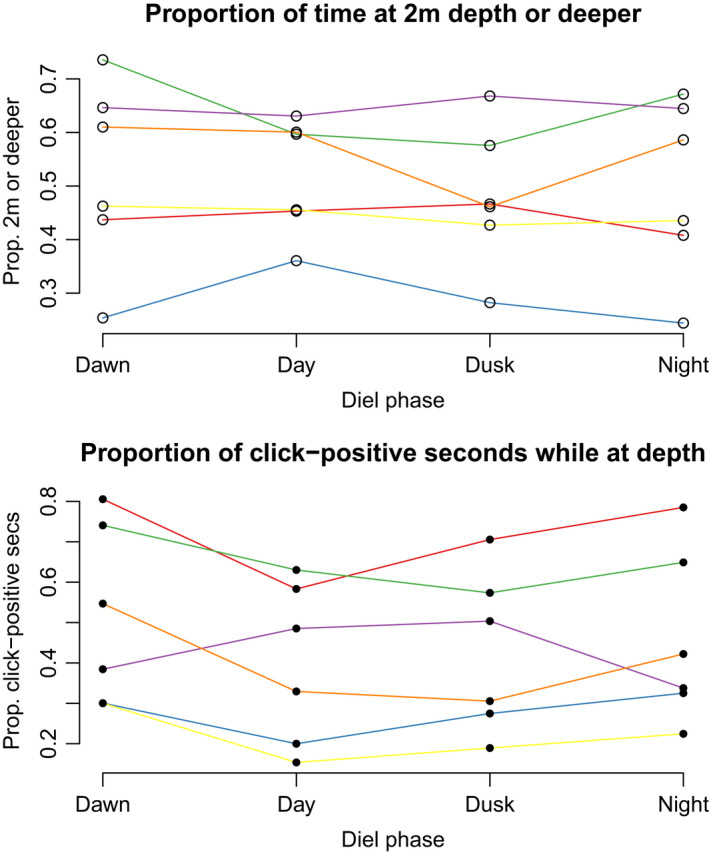
Top panel: proportion of time spent at depths of 2 m or deeper by diel phase for 6 tagged harbour porpoise. Bottom panel: proportion of click‐positive seconds by diel phase for 6 tagged harbour porpoise; in this plot, only records where the tag was at depth of 2 m or deeper were used

**FIGURE A3.2:**
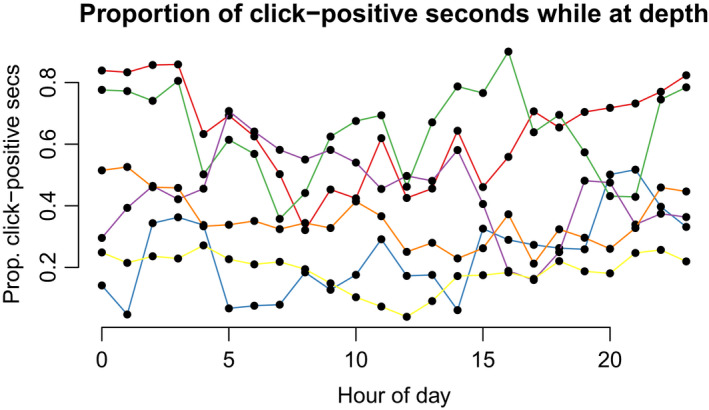
Proportion of click‐positive seconds by hour of day (coordinated universal time, UTC) for 6 tagged harbour porpoise; in this plot, only records where the tag was at depth of 2 m or deeper were used

## Data Availability

All processed data and R code files to reproduce the results given in this paper are uploaded to Dryad, doi: https://doi.org/10.5061/dryad.n5tb2rbx7.

## APPENDIX 1

### Field calibration of C‐PODs

The playback data recorded in the main survey area (see Playback experiment in the paper) were also used to evaluate the C‐PODs performance, by estimating their detection threshold. The threshold was defined as the average received level of the artificial harbour porpoise‐like clicks in a playback, when 50% of the transmitted clicks in a click sequence were logged. Each sequence consisted of 10 or 20 clicks of the same source level. The C‐POD measures the sound pressure level (SPL) of each click as maximum peak‐to‐peak range and logs it on an 8‐bit scale, which can be converted to SPL values in Pascal (Tregenza, 2014). After identifying the SPL of the click sequence with 50% of the clicks logged, or interpolated the SPL between the two click sequences closest above and below the 50% threshold, the SPL was converted to dB re 1 µPa. Overall average for 58 selected C‐PODs was 117.6 dB re 1 µPa (SD 1.2 dB) (Figure A1.1).

A subset of nine of the C‐PODs included in the field calibration analysis were also calibrated in a tank. Their average threshold was 118.1 dB re 1 µPa (SD 2.4 dB) using the playback data and 116.4 dB re 1 µPa (SD 4.2 dB) using the tank data. The results are comparable to the published literature (Dähne, Gilles, et al., 2013), taking into account that the field calibrations were carried out in a more variable acoustic environment than calibrations carried out in a tank. The tight standardization of the C‐PODs ensures that the data collected by all loggers result in comparable field recordings given the same acoustic conditions.

## APPENDIX 2

### False‐positive rate

We processed the data first through the KERNO classifier and then through the Hel1 classifier. The Hel1 classifier was developed from C‐POD data on porpoises in Polish waters collected by the Hel Marine Station and analyzed in a two‐day workshop by six analysts in Hel, prior to this project. The Hel1 classifier aims to distinguish porpoise clicks from other sources of clicks resembling porpoise clicks, occurring in the Baltic Sea. It identifies “encounters” that are separated by at least 10 min without click trains categorized as narrow‐bandwidth high‐frequency species (NBHF; in practice harbour porpoises, for deployments in the Baltic Sea) by the KERNO classifier. It then classifies the aggregated click train features within each encounter. It assumes that there are no dolphins present, which is likely to be a valid assumption for the Baltic Sea as the harbour porpoise is the only resident species of cetacean. Highly atypical encounters, such as those consisting of only one click train, or only very weak trains with very short inter‐click intervals, or only trains that correspond in time to a burst of lower frequency noise, are rejected.

For estimation of the false positive rate, it was assumed that the spatial and temporal distribution of false positives was unrelated to porpoise detections and that false positives were randomly distributed in time and space. The SAMBAH data comprised a total effort of 1343 files with a total duration of 377 years and 25 days. Of these, 359 files had an average detection rate of <60 detection positive minutes (DPM) per year. The total recording effort of this subset was 40,726 days. As these files were most likely to have the highest rate of false positives, they were selected for an evaluation of the performance of the Hel1 classifier with regard to the prevalence of false positives.

A two‐day workshop was held in which 12 trained analysts visually inspected the selected files in the custom C‐POD software that provides views of the timing, amplitude, frequency, duration, and bandwidth of clicks on a wide range of time scales. The analysts applied wider criteria of validity not used in the Hel1 classifier, such as the presence of fragments of trains of long, not‐weak, porpoise‐like clicks before or after the defined encounter, as these are usually seen in actual porpoise encounters. The visual validation was carried out before all files were truncated to start at midnight after deployment and end at midnight before retrieval, with a margin of at least one hour. The truncation was done at a later stage to discard most playbacks not already rejected by Hel1 in the entire dataset. With very few exceptions, the playbacks were carried out in conjunction with deployment or retrieval.

The likely origin of the false detections was noted for a subset of 200 visually validated files. These files contained a total of 176,000 DPM classified as harbour porpoise by Hel1, whereof 157 DPM were determined as false positives by the analysts. The analysists found that the most common source of false positives was boat sonars (76 DPM), followed by playback experiment signals (65 DPM), weak unknown train sources (10 DPM), and unidentified non‐porpoise signals (6 DPM).

The total duration of the subset of 200 files was 22,698 days. This gives a rate of 1 false DPM per 145 recording days. Assuming the same rate of false positives in the non‐inspected files, the total number of non‐removed false DPM in the entire dataset was 671, not taking into account that playbacks were largely removed from these files by truncation. Taking into account that most playbacks were omitted from the dataset by truncation of the files at midnight, the rate of false positives in the non‐inspected dataset was more likely to be around 1 false DPM per 247 recording days, or 393 false DPM in the entire dataset. Our conclusion is that the observed rate of false positives had a negligible impact on the abundance estimations, far below other sources of error.

## APPENDIX 3

### Diel patterns in detectability and click production

In our main analyses, we found diel variation in estimated effective detection area (EDA) of porpoises in the Great Belt tracking experiment and in the acoustic detection rates of porpoises (click‐positive seconds (CPS) per thousand seconds of survey effort) in the main survey (Table 1). We found that, relative to day, the estimated porpoise EDA in the tracking experiment was 26.4 times higher at night, 3.2 times higher at dusk and 1.9 times higher at dawn. By contrast, in the main survey, we found much less variation, and a different ordering of dusk and dawn: relative to day detection rates were 2.1 times higher at night, 1.2 at dusk, and 1.4 at dawn. In the discussion, we speculate that these diel differences may be due to diel changes in acoustic behavior or possibly propagation/masking.

Manuscript reviewers suggested that there may be information about diel changes in acoustic behavior in the tags used in the tagging, and about diel variation in click detectability in the playback experiments performed both in the Great Belt and the main study area. In this Appendix, we undertake preliminary investigations of these datasets.

### Diel variation in click production from tag records

#### Methods

For each of the 6 tagged porpoises, we divided the acoustic record by diel phase, and for each phase, we calculated the proportion of seconds of recording in which clicks were recorded (proportion of CPS). As noted in the main paper, records from when the porpoise was at depths shallower than 2 m are too noisy to use, and so we only used seconds when the tag was deeper than 2 m in this analysis. In case diel patterns of time spent near the surface affected our results (and as a crude measure of diel changes in diving behavior), we also calculated the proportion of time spent deeper than 2 m by diel phase. Lastly, in case examining the data by diel phase was obscuring any pattern, we also looked at proportion of CPSs by hour of the day.

#### Results

The tags were deployed in March (1 tag), April (1 tag), May (2 tags), and July (2 tags), and at those times of year, the majority of time overall was in diel phase day. On average, the percentage of tag records from dawn, day, dusk, and night was 4%, 65%, 4%, and 28%, respectively. Hence, results for day and night will be more reliable than those for dawn and dusk.

Although the 6 animals showed large between‐animal variation in the proportion of time spent at 2 m or greater depth (from 0.39 to 0.65), there was relatively little within‐animal variation by diel phase and no consistent pattern in this across animals (Figure A3.1 top panel). The mean (averaging across animals, not applying any weighting for the number of records per tag) for dawn, day, dusk and night was 0.52, 0.51, 0.48, and 0.50.

There was also large between‐animal variation in proportion of CPS, ranging from 0.22 to 0.72. There was some within‐animal variation by diel phase, but it was not consistent across animals (Figure A3.1 bottom panel). Four animals had a higher proportion of CPSs in night than day, one lower and one about the same. Patterns for dawn and dusk relative to day were even more mixed. Overall, the mean (across animals, unweighted) proportion of CPSs for dawn, day, dusk, and night was 0.51, 0.40, 0.43, and 0.45. This gives an average of 15% more CPSs in the night phase vs day and 28% more in dawn vs day. Proportion of CPSs by hour of day likewise showed no consistent pattern across animals (Figure A3.2).

### Diel variation in estimated effective detection area from playbacks made during great belt tracking study

In the tracking study, playbacks were only performed during daylight hours: The earliest was at 10:21 and the latest 16:35 (UTC). Hence, these data cannot be used to address questions of diel variation in click detectability.

### Diel variation in estimated effective detection area from playbacks made in the main survey area

#### Methods

In the main paper, a binary generalized additive model (GAM) including environmental, temporal, and spatial variables was fitted to the playback experiment data. The fitted model, which explained 54.9% of the deviance, was used to predict EDA at all main survey sites and months. Here, we added diel phase as a 4‐level factor variable. With the new model, we predicted EDA at all main survey sites and months for each diel phase, and examined the differences between diel phases.

#### Results

The GAM including diel phase had a lower AIC than that without this factor, but only a slightly greater percentage of the deviance was explained by including diel phase (1% more, at 55.9%). Model coefficients were also relatively small: −0.43, −0.22, and 0.59 for dawn, dusk, and night, respectively (day was coded as the baseline level). These coefficients can be interpreted as the log odds ratio for that factor level relative to the baseline, so exponentiating them gives the log odds—in other words the odds of detecting a click from the playbacks at dawn, dusk, and night relative to the odds in the day are 0.64, 0.80, and 1.80, respectively. If detectability was higher at dawn and dusk relative to day, we would expect the odds ratio to be more than 1, not <1.

The results perhaps are made more interpretable by looking at the change in estimated mean playback EDA, where the mean is taken over all stations and months. The estimates for dawn, day, dusk and night are 15.0, 19.3, 17.1, and 26.5 ha, respectively. Dividing by the value for day gives estimates of relative playback EDA for dawn, dusk, and night of 0.78, 0.89, and 1.39, respectively.

### Summary, discussion and conclusions

In the Great Belt study, we estimated porpoise EDA to be over 20 times higher at night than day, and 2–3 times higher at dusk and dawn. From the main survey, we obtained just over 2 times the detections at night vs day, and 1.2–1.4 times as many at dusk and dawn. Of the two “shoulder” periods, dusk was higher in the Great Belt study and dawn in the main survey.

Here, we found diel variation in click production (measured as CPSs) from six tagged porpoises, but no consistent patterns. Averaging over the porpoises, there was a slight tendency for more CPSs at night than the day, but the value was even higher at dawn which does not match the patterns described in the previous paragraph. We conclude that, from this small sample of animals, there is no evidence for consistent diel variation in acoustic behavior causing the observed patterns in detections in the main survey. This is broadly in line with the finding of Linnenschmidt et al. (2013) , who found large differences in acoustic behavior between three harbour porpoises tagged in Danish waters (for shorter time period than those used here), and varying diel patterns (see also Wisniewska et al. (2018) for another example). We note that our data come from spring and summer, and hence, we can make no inferences about what might happen in the months where night‐time predominates. Lastly, we were not able to investigate the acoustic record; however, we note that Macaulay (2020) found variation in click source level was predicted to be a major factor influencing detectability and so diel variation in source level would be particularly interesting in investigate in future.

Analysis of the playback experiments that took place throughout the study area did appear to reveal small changes in detectability of artificial porpoise clicks between with diel periods. Detectability was estimated to be 1.4 times higher at night than day, but was lower at dawn and dusk. Hence, diel variation in detectability may go some way to explain the observed increase in detection in the main study at night compared with day, but does not explain the dawn and dusk patterns. One possibility is that there is general diel variation in anthropogenic or other interfering noise although none was noted during the playback experiments; another is variation in the physical environment causing changes in propagation, although we are not aware of a plausible mechanism. This topic warrants further investigation, for example, to examine more closely whether the patterns in playback results may be stronger in particular parts of the study area and/or seasons. We could also examine whether the diel patterns in detection rates in the main survey vary by location and/or season. A year‐long study by Schaffeld et al. (2016) at 5 sites in the western Baltic found that diel patterns varied between sites and seasons. An examination of part of the SAMBAH dataset for the presence and frequency of foraging events (indicated by high repetition‐rate “buzz” clicks) showed higher incidence at dawn and night, with possible regional variation (Kyhn et al., 2018).

We were not able to examine potential diel changes in detectability in the Great Belt site because we only undertook playback experiments in the daytime. Future studies should consider whether diel variation may be a factor and, if so, undertake playbacks in all diel phases.

Overall, these preliminary examinations have not been able to fully explain the diel patterns in detection found at Great Belt and the main study area. This points to the need for further research on diel variation in acoustic behavior and detectability. We thank the reviewers for prompting us to undertake these additional studies.

## APPENDIX 5

**TABLE A5.1:** Variables considered for inclusion in the analysis of playback experiments in the main survey area

Variable	Order of inclusion^a^	Notes^b^
Distance^c^	1	Distance (m) from the device to the transducer
SL_p‐p_ ^c^	1	Peak‐to‐peak source level (dB re 1 µPa m) of the click
Depth	2	Water depth (m) at station
SST	2	Sea surface temperature (^o^C)
SBT	2	Sea bottom temperature (^o^C)
Sssal	2	Sea surface salinity (PSU)
SBsal	2	Sea bottom salinity (PSU)
Pdepth	2	Pycnocline depth (m)
Pgrad	2	Pycnocline gradient (kg/m^3^/m)
Geo	2	Sediment type. Factor covariate, with 5 levels: mud to sandy mud; sand to muddy sand; coarse‐grained sediment; mixed sediment; till, boulders and bedrock.
Year	3	Year (1–2)
Month	3	Month (1–12) when experiment took place
Day	3	Julian day (i.e. 1 = 1st Jan, 2 = 2nd Jan, …., 365 = 31 Dec)
Lon^d^	3	Longitude of station (^o^E)
Lat^d^	3	Latitude of station (^o^N)

^a^ Variables associated with playback (denoted 1) were offered for inclusion in the forward model selection algorithm first, followed by those associated with the acoustic environment (denoted 2). Lastly, temporal and spatial variables (denoted 3) were offered. Since month and day are confounded, then once either month or day was included, the other was not considered.

^b^ Variables were included as continuous smooths except for Geo, which was a factor covariate.

^c^ The interaction (tensor product) between Distance and SL_p‐p_ was also offered.

^d^ The interaction (tensor product) between Lon and Lat was also offered.

**TABLE A5.2:** Estimated probability of one or more click‐positive seconds (p(click)) during a tracking event in the acoustic tag data in the tracking experiment

Harbour porpoise	p(click)	Recording duration (s)
1	0.958	60,082
2	0.671	41,922
3	0.936	18,198
4	0.941	22,615
5	0.851	52,313
6	0.708	72,151
Weighted mean	0.822 (SE 0.056)	

Note

Data for six tagged harbour porpoises and weighted mean across them, weighting by recording duration. Recording duration is the total number of seconds the tag was recording at depth >2 m. The tags were duty cycled (typically recording 10 min per hour), so the recordings were done over a longer period of time.

**TABLE A5.3:** Summary of playback data by country in the main survey area

Country	Number of survey stations	Number of survey stations with playback experiments	Number of playback experiments	Mean number of distances per playback experiment	Mean min. and mean max. distance per playback experiment (m)
Denmark	21	16	36	3.5	211–250
Estonia	40	0	0	–	–
Finland	46	25	25	4.2	51–226
Germany	16	16	32	6.5	173–357
Latvia	34	9	12	2.9	123–170
Lithuania	9	6	10	2.8	80–125
Poland	39	39	68	4.0	351–500
Sweden	99	70	70	3.5	68–190
Total	304	181	253		
Mean				4.0	113–303

**TABLE A5.4:** Summary of playback data by month in the main survey area

Month	Number of playbacks
January	0
February	8
March	15
April	27
May	89
June	25
July	2
August	48
September	0
October	17
November	21
December	1
Total	253

**TABLE A5.5:** Estimates and standard error (SE) for parametric coefficients in the model of playback experiments in the main survey area

Coefficient	Estimate	Standard error
Intercept (mud to sandy mud)	−5.781	0.792
Sand to muddy sand	0.436	0.022
Coarse‐grained sediment	0.996	0.292
Mixed sediment	1.111	0.026
Till, boulders and bedrock	1.001	0.038

Note

The parametric coefficients relate to the sediment type factor covariate. All coefficients have *p* < 2E‐13 in a *z*‐test of the null hypothesis that the coefficient is zero.

**TABLE A5.6:** Estimates of density and abundance of harbour porpoises in the main survey area during May–October at the level of country within region

Country	Region	Area (km^2^)	Density (animals/1000 km^2^)		Abundance		CV (%)
			Estimate	95% CI	Estimate	95% CI	
Denmark Bornholm	Northeast	848	0	–	0	–	–
Estonia	Northeast	24,588	0	0–0	0	0–0	–
Finland	Northeast	23,005	0	0–0	0	0–0	–
Latvia	Northeast	18,735	0.10	0–0.23	2	0–4	69.1
Lithuania	Northeast	5719	0	0–0	0	0–0	–
Poland	Northeast	14,849	0.48	0.07–0.96	7	1–14	53.9
Sweden	Northeast	44,860	10.74	1.41–24.36	482	63–1093	69.1
Denmark excl. Bornholm	Southwest	3797	3039.14	1386.81–5795.17	11,538	5265–22,002	36.7
Denmark Bornholm	Southwest	7932	1.63	0.15–4.26	13	1–34	70.3
Germany	Southwest	8412	593.57	250.92–1214.57	4993	2111–10,216	40.8
Poland	Southwest	7092	5.18	0.66–11.74	37	5–83	55.8
Sweden	Southwest	6750	674.87	118.96–1720.40	4555	803–11,612	65.7

Note

CI = confidence interval; CV = coefficient of variation. Northeast = Within the May–October distribution range of the Baltic Proper porpoise population as defined by Carlén et al. (2018); southwest = From the Darss and Limhamn/Drogden sills in the west to the May–October border of the Baltic Proper porpoise population as defined by Carlén et al. (2018) in the east.

**TABLE A5.7:** Estimates of density and abundance of harbour porpoises in the main survey area during November–April at the level of country within region

Country	Region	Area (km^2^)	Density (animals/1000 km^2^)		Abundance		CV (%)
			Estimate	95% CI	Estimate	95% CI	
Denmark Bornholm	Northeast	848	2.40	–	2	–	–
Estonia	Northeast	24,588	0	0–0	0	0–0	–
Finland	Northeast	23,005	0.55	0.08–2.38	13	2–55	112.7
Latvia	Northeast	18,735	0.49	0.01–1.96	9	0–37	114.7
Lithuania	Northeast	5719	1.46	0.04–3.98	8	0–23	79.5
Poland	Northeast	14,849	1.53	0.57–3.14	23	9–47	43.5
Sweden	Northeast	44,860	4.19	1.15–10.01	188	52–449	62.8
Denmark excl. Bornholm	Southwest	3797	2,126.32	823.98–4998.17	8,073	3128–18,976	51.0
Denmark Bornholm	Southwest	7932	10.08	1.00–28.96	80	8–230	76.1
Germany	Southwest	8412	261.85	89.66–617.08	2,203	754–5191	51.2
Poland	Southwest	7092	2.31	0.44–6.69	16	3–47	73.7
Sweden	Southwest	6750	54.56	21.11–127.88	368	143–863	49.7

Note

CI = confidence interval; CV = coefficient of variation. Region: northeast = within the May–October management range of the Baltic Proper porpoise population as defined by Carlén et al. (2018); southwest = from the Darss and Limhamn/Drogden sills in the west to the May–October management border of the Baltic Proper porpoise population as defined by Carlén et al. (2018) in the east.

**TABLE A5.8:** Offshore wind farms in all stages from concept to pre‐construction within the northeast and southwest regions of the main survey area

Region	Stage	DK	EE	FI	LT	LV	PL	SE	DE	Sum
Northeast	Concept/early planning		4	1	1		18	8		32
Northeast	Consent application submitted		1		2			2		5
Northeast	Consent authorized						2			2
Northeast	Pre‐construction									0
Northeast	Sum northeast	0	5	1	3	0	20	10	0	39
Southwest	Concept/early planning	1					7	3	1	12
Southwest	Consent application submitted									0
Southwest	Consent authorized							1	4	5
Southwest	Pre‐construction	1							1	2
Southwest	Sum southwest	2	0	0	0	0	7	4	6	19
	Total sum	2	5	1	3	0	27	14	6	58

Note

Data from 4COffshore (2020). Region: northeast = within the May–October management range of the Baltic Proper porpoise population as defined by Carlén et al. (2018); southwest = from the Darss and Limhamn/Drogden sills in the west to the May–October management border of the Baltic Proper porpoise population as defined by Carlén et al. (2018) in the east.
